# Plant hormone and peptide signaling converge in the genetic network regulating cambium activation in Arabidopsis roots

**DOI:** 10.1093/plcell/koag059

**Published:** 2026-03-13

**Authors:** Tiina Blomster, Riccardo Siligato, Riikka Mäkilä, Wiktoria Fatz, Munan Lyu, Maria Angels de Luis Balaguer, Lingling Ye, Iris Gildea, Omid Safronov, Panu Somervuo, Kamil Růžička, Jarkko Salojärvi, Rosangela Sozzani, Melis Kucukoglu Topcu, Ari Pekka Mähönen

**Affiliations:** Department of Organismal and Evolutionary Biology, Faculty of Biological and Environmental Sciences, and Viikki Plant Science Centre, University of Helsinki, Helsinki 00014, Finland; Department of Organismal and Evolutionary Biology, Faculty of Biological and Environmental Sciences, and Viikki Plant Science Centre, University of Helsinki, Helsinki 00014, Finland; Department of Organismal and Evolutionary Biology, Faculty of Biological and Environmental Sciences, and Viikki Plant Science Centre, University of Helsinki, Helsinki 00014, Finland; Department of Organismal and Evolutionary Biology, Faculty of Biological and Environmental Sciences, and Viikki Plant Science Centre, University of Helsinki, Helsinki 00014, Finland; Department of Organismal and Evolutionary Biology, Faculty of Biological and Environmental Sciences, and Viikki Plant Science Centre, University of Helsinki, Helsinki 00014, Finland; Department of Plant and Microbial Biology, North Carolina State University, Raleigh, NC 27695, United States; Department of Organismal and Evolutionary Biology, Faculty of Biological and Environmental Sciences, and Viikki Plant Science Centre, University of Helsinki, Helsinki 00014, Finland; Department of Organismal and Evolutionary Biology, Faculty of Biological and Environmental Sciences, and Viikki Plant Science Centre, University of Helsinki, Helsinki 00014, Finland; Department of Organismal and Evolutionary Biology, Faculty of Biological and Environmental Sciences, and Viikki Plant Science Centre, University of Helsinki, Helsinki 00014, Finland; Faculty of Biological and Environmental Sciences, University of Helsinki, Helsinki 00014, Finland; Department of Organismal and Evolutionary Biology, Faculty of Biological and Environmental Sciences, and Viikki Plant Science Centre, University of Helsinki, Helsinki 00014, Finland; Laboratory of Hormonal Regulations in Plants, Institute of Experimental Botany, The Czech Academy of Sciences, Prague 160 00, Czech Republic; Department of Organismal and Evolutionary Biology, Faculty of Biological and Environmental Sciences, and Viikki Plant Science Centre, University of Helsinki, Helsinki 00014, Finland; School of Biological Sciences, Nanyang Technological University, Singapore 637551, Singapore; Department of Plant and Microbial Biology, North Carolina State University, Raleigh, NC 27695, United States; Department of Organismal and Evolutionary Biology, Faculty of Biological and Environmental Sciences, and Viikki Plant Science Centre, University of Helsinki, Helsinki 00014, Finland; Department of Organismal and Evolutionary Biology, Faculty of Biological and Environmental Sciences, and Viikki Plant Science Centre, University of Helsinki, Helsinki 00014, Finland

## Abstract

Plant secondary growth is driven by 2 concentric meristems: the inner vascular cambium and outer cork cambium. The periclinal cell divisions of both meristems, providing thickness and protection to plant organs, are activated with a delay after the primary development. Cytokinins and a set of downstream transcription factors are key players in promoting transition from primary to secondary development; however, it is unknown whether other factors play a role in this transition. Here, using time-course transcriptome analysis of cytokinin-treated, cytokinin-deficient *isopentenyltransferase1,3,5,7* (*ipt1,3,5,7*) mutant, we show that during cambium activation cytokinins positively regulate auxin and TRACHEARY ELEMENT DIFFERENTIATION INHIBITORY FACTOR (TDIF) peptide signaling in Arabidopsis (*Arabidopsis thaliana*) root. Correspondingly, mutants defective in TDIF peptide signaling displayed reduced cytokinin-induced secondary growth, and auxin signaling was found to be required for proper cytokinin response. Additionally, auxin and cytokinin signaling transiently overlapped in activating procambial cells and acted additively in promoting secondary development. Network analysis revealed that transcription factors belonging to the DNA-BINDING WITH ONE FINGER (DOF) and ETHYLENE RESPONSE FACTOR (ERF) gene families, are regulated by cytokinin during cambium activation and mutant analysis demonstrated delayed cambium activation and xylem formation phenotypes. Overall, we find that cytokinin, auxin, and TDIF form a tightly intertwined network of positive regulators for activation of secondary growth in the Arabidopsis root, indicating extensive redundancy in this process.

## Teaser

An intertwined network of cytokinin, auxin and peptide hormone signaling activates secondary growth in the Arabidopsis roots.

## Introduction

Plant secondary growth ensues from the activity of secondary meristems called vascular cambium and cork cambium. These meristems contain stem cells, which divide periclinally, and the daughter cells differentiate into either vascular (from vascular cambium) or protective (from cork cambium) cell types (Evert 2006; Wybouw et al. 2024). The importance of plant hormone cytokinin for secondary vasculature formation and subsequent secondary growth has been shown in multiple species, especially in Arabidopsis (*Arabidopsis thaliana*) (Matsumoto-Kitano et al. 2008; Randall et al. 2015; Ye et al. 2021), but also in radish (*Raphanus sativus*) (Jang et al. 2015) and hybrid aspen (*Populus tremula x tremuloides*) (Nieminen et al. 2008; Immanen et al. 2016). Transcriptional response to cytokinin is mediated by Arabidopsis type-B response regulators (ARRs) (Argueso and Kieber 2024). Type-B ARRs directly activate the transcription of *LATERAL ORGAN BOUNDARIES* (*LOB*) *DOMAIN-CONTAINING PROTEIN* (*LBD*) transcription factors *LBD3/ASYMMETRIC LEAVES 2-LIKE 9* (*ASL9*) and *LBD4* during cambium activation, and *LBD3* and *LBD4* are both necessary and sufficient for activation of the secondary growth (Ye et al. 2021). Cell divisions in vascular cambium are promoted by TDIF-PXY (TRACHEARY ELEMENT DIFFERENTIATION INHIBITORY FACTOR—PHLOEM INTERCALATED WITH XYLEM) receptor-ligand interaction, in part through transcription factors *WUSCHEL-RELATED HOMEOBOX 4* (*WOX4*) and *WOX14* (Ito et al. 2006; Fisher and Turner 2007; Hirakawa et al. 2008, 2010; Etchells and Turner 2010; Etchells et al. 2013) as well as through CAMBIUM EXPRESSED AINTEGUMENTA-LIKE (CAIL) transcription factors (Eswaran et al. 2024).

Auxin's importance in regulating secondary growth has been shown in both Arabidopsis (Suer et al. 2011; Brackmann et al. 2018 ; Smetana et al. 2019; Mäkilä et al. 2023; Zhang et al. 2025) and hybrid aspen (Nilsson et al. 2008). Importantly, auxin maximum in the xylem domain defines the organizer for the bifacial cambial stem cells (Smetana et al. 2019). However, auxin signaling has also been reported as a negative regulator of cambium activity in Arabidopsis stem (Brackmann et al. 2018), and therefore the role of auxin during secondary growth may be dependent on the spatiotemporal dynamics or developmental context. The interaction of auxin and cytokinin signaling has been shown to govern plant primary growth both in the root and shoot apical meristems (RAM and SAM, respectively). Typically, cytokinin and auxin act antagonistically and occupy adjacent domains to drive morphogenesis (Schaller et al. 2015; De Rybel et al. 2016; Wang et al. 2023). In the procambium of RAM, auxin and cytokinin form a mutually antagonistic signaling feedback loop. This loop involves the cytokinin signaling inhibitor ARABIDOPSIS THALIANA HISTIDINE PHOSPHOTRANSFER PROTEIN 6 (AHP6) and polar auxin PIN-FORMED (PIN) transporters, which operate in distinct, non-overlapping regions (Mähönen et al. 2006; Bishopp et al. 2011). Within the xylem pole pericycle lineage in the root, auxin signaling promotes lateral root specification, while cytokinin signaling favors cambial identity (Wang et al. 2025). In the SAM, expression of cytokinin signaling primary targets and negative regulators, type-A RRs *ARR7* and *ARR15*, is decreased by auxin (Zhao et al. 2010). In the vascular cambium PXY-dependent phosphorylation of AUXIN RESPONSE FACTOR 5/MONOPTEROS (ARF5/MP) downregulates cytokinin signaling, resulting in reduced vascular cambium activity (Han et al. 2018). However, it is unknown how cytokinin and auxin signaling interact during the activation of secondary growth.

Here, we show that, unlike in the primary meristems, auxin and cytokinin signaling transiently overlap and co-operatively activate the vascular cambium in Arabidopsis roots. We find that cytokinins induce auxin signaling, and auxin responsiveness is required for proper cytokinin-mediated induction of cambium activity. Time-course gene expression analysis revealed that also TDIF peptide signaling is positively regulated by cytokinin treatment. The identification of cambial regulators from our network analysis and their connections to hormonal and peptide signaling pathways will help to understand the complex signaling at the onset of secondary growth.

## Results

### Identification of cytokinin-induced gene expression at the onset of cambium activation

**Figure 1:**
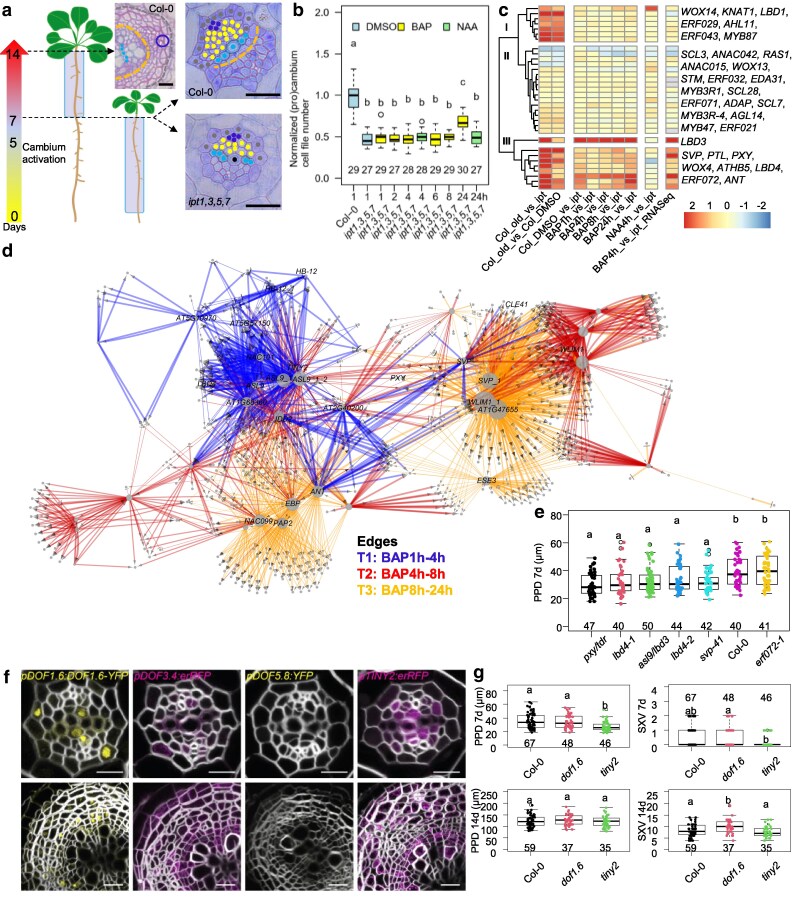
Transcriptomic network regulating secondary growth. **a)** Secondary growth activation in the Arabidopsis root. Histological cross-sections of wild-type Col-0 14-d-old root, 7-d-old Col-0 root, and 7-d-old *ipt1,3,5,7* mutant root. Colors represent primary xylem (light blue = differentiated, black = undifferentiated), (pro)cambial cells (yellow), vascular cambium (orange), primary phloem (dark blue), and pericycle (gray). Scale bar = 20 µm. Blue boxes represent main root sections harvested for gene expression time series experiment. **b)** 7-d-old *ipt1,3,5,7* mutant roots were treated with DMSO (1 h), 1 µM BAP (1, 2, 4, 6, 8, or 24 h), or 1 µM NAA (4 or 24 h). Procambial cell file numbers were quantified from histological cross sections and normalized to median of 7-d-old wild-type Col-0 treated with DMSO 1 h. The experiment was performed in triplicate and followed by RNA extraction from the main roots as indicated in a). **c)** Hierarchical clustering of known cambial transcription factors and secondary growth regulators in the microarray (Agilent 4 × 44 K) and RNA-Seq data sets. Mean values of log_2_ fold changes calculated from gene-specific probes are shown. Accession numbers are listed in Supplementary Data Set S1. **d)** Gene regulatory network of cytokinin-induced cambium activation. Genes clustering with *ANT* were depicted according to their temporal cytokinin response (T1, T2, and T3). The node edges represent a regulatory connection, the node size represents the number of connections, while the edge colors indicate the first increase in expression during the time-point comparisons. For clarity, only names of selected genes are shown, and nodes without edges were omitted. **e)** Protophloem distance (PPD) of 7-d-old mutants of known cambial factors in the GRN and wild-type Col-0 were analyzed from histological cross sections. Experiment was repeated 3 times. **f)** Root cross sections of translational reporter line *pDOF1.6:DOF1.6-YFP* and transcriptional reporter lines *pDOF3.4:erRFP*, *pDOF5.8:YFP* and *pTINY2:erRFP* from 5-d-old (upper row, scale bar 10 µm) and 14- to 16-d-old (lower row, scale bar 20 µm) plants. **g)** Protophloem distance (PPD) and secondary xylem vessel (SXV) numbers of Col-0, *dof1.6*, and *tiny2* roots (upper row: 7 d, lower row: 14 d). The experiment was repeated 4 times. In b, e, and g numbers depict number of roots analyzed with Fiji (n), the boxes in the box-and-whisker plots show the median and interquartile range, the whiskers indicate 1.5 × the interquartile range, and outliers are shown as circles. Letters indicate statistical significance following Wilcoxon rank sum test (adjusted *P*-value < 0.05). In e and g, individual data points are plotted on top of the box-and-whisker plots.

Cambium activation defined by initiation of periclinal cell divisions in procambial and pericycle cells takes place in the upper part of 5-d-old wild-type Arabidopsis roots in a cytokinin-dependent manner (Matsumoto-Kitano et al. 2008; Ye et al. 2021; Wang et al. 2023; Shimadzu et al. 2025), and secondary development continues indeterminately as the root matures (Fig. 1a). To study the cytokinin-induced secondary growth at the activation stage, we treated the *isopentenyltransferase* (*ipt*) *1,3,5,7* mutant, which is unable to initiate secondary growth in the root (Matsumoto-Kitano et al. 2008), with cytokinin time series (1, 2, 4, 6, 8, and 24 h 6-benzyladenopurine; BAP) (Fig. 1b) (Randall et al. 2015). Auxin treatment time points (4 and 24 h 1-naphthaleneacetic acid; NAA) were also included to study the role of auxin in cambium activation (Fig. 1b). Increase in procambial and pericycle cell numbers marking secondary growth initiation was observed after 24-h BAP treatment (Fig. 1b; Fig. S1). In contrast, the auxin treatments were insufficient to rescue the *ipt1,3,5,7* vascular cambium cell divisions, but 24-h NAA treatment increased pericycle cell numbers (Fig. S1), which is likely associated with auxin-induced periderm or lateral root formation (Parizot et al. 2008; Xiao et al. 2020).

Since our established cytokinin-treatment timeline captures increased expression of cambial marker genes *AINTEGUMENTA* (*ANT*) and *CYCLIN D3;1* (*CYCD3;1*) before cambial cell divisions (Randall et al. 2015), we profiled the genome-wide transcriptional secondary growth activation of 7-d-old *ipt1,3,5,7* seedlings (1, 4, 8, and 24 h BAP) with Agilent 4 × 44 K microarrays and RNA-Seq (4 h BAP). Additionally, 14-d-old Col-0 root samples with advanced secondary growth, 7-d-old Col-0 roots with early secondary growth and 7-d-old 4-h NAA–treated *ipt1,3,5,7* roots were included to identify genes consistently expressed in different developmental stages and specifically regulated by cytokinin, respectively (Fig. 1a, b). Transcriptomic differences were calculated in comparison to the *ipt1,3,5,7* 1 h DMSO control and between 7-d and 14-d Col-0 samples (Supplementary Data Set S2). Hierarchical clustering of 33 known cambial factors (Zhang et al. 2019) identified 3 main clusters in our data set (Fig. 1c). Cluster III genes (*ANT*, *LBD4*, *ASL9/LBD3*, *PXY*, *WOX4*, *PETALLOSS* (*PTL*), *SHORT VEGETATIVE PHASE* (*SVP*), *ETHYLENE RESPONSE FACTOR 072* (*ERF072*), and *ARABIDOPSIS THALIANA HOMEOBOX PROTEIN 5* (*ATHB5*)) were cytokinin-induced already at 4 h RNA-Seq analysis (log_2_FC ≥ 0.5, adjusted *P*-value ≤ 0.05), well before the observed cambial cell divisions (Fig. 1b, c; Supplementary Data Set S2). Cluster I and cluster III transcripts were more expressed in 14-d-old roots compared with 7-d-old roots, therefore reflecting the progression of secondary growth, while cluster II transcripts lacked major changes in expression (Fig. 1c). These results indicate selectivity in the transcriptional responses of the cambial genes in this experimental set-up.

### Transcriptional network during cambium activation

Next, we set out to identify key regulators of cambium activation from our transcriptomic data set. Thus, we decided to infer the transcriptional network of cambium activation by using the GENIST method (De Luis Balaguer et al. 2017). By interrogating genes coexpressed with cambial marker *ANT*, we obtained a gene regulatory network (GRN) of 65 nodes representing microarray probes for 56 transcription factors and more than 400 genes/probes altogether (Fig. 1d; Supplementary Data Set S3; Fig. S2; see Materials and Methods for details). To validate the network, we studied 7-d-old mutant lines of known cambial factors present in the network and observed reduced vascular diameter (ie protophloem distance, Fig. S1) in the *pxy/tdr* (Hirakawa et al. 2010), *lbd4-1*, *lbd4-2*, *asl9/lbd3* (Zhang et al. 2019), and *svp-41* (Gregis et al. 2006) lines (Fig. 1c, e; Fig. S3). We also observed a time-dependent enrichment in our network, with cytokinin responsive (Bhargava et al. 2013) and primary cytokinin response genes (ARR10 targets) (Zubo et al. 2017) showing statistically most significant enrichment at early and cambium-enriched genes (Zhang et al. 2019) at later stages (Fig. S2). This suggests that the GRN likely reflects transcriptional activation of the cambium induced by cytokinin. Correspondingly, nodes representing the early cytokinin-responsive cambial regulator *LBD3* (*ASL9*) exhibited a large number of edges in T1 (1 to 4 h) and T2 (4 to 8 h) comparisons, but no edges at T3 (8 to 24 h) (Supplementary Data Set S3; Fig. S2). *LBD4* showed connections only with its homolog LBD3 (Supplementary Data Set S3). Other transcription factors (TFs) with edges emerging predominantly at early time points included *AT2G40200* and *AT5G57150* encoding basic helix-loop-helix (bHLH) DNA-binding superfamily proteins, *INDETERMINATE DOMAIN2* (*IDD2*), *NAC-domain protein101* (*NAC101*)/*VASCULAR-RELATED NAC-DOMAIN6* (*VND6*), *TINY2* (*AT5G11590*), *AT1G68360* (*GLABROUS INFLORESCENCE STEMS3* (*GIS3*)), *AT5G10970* (C2H2 and C2HC zinc fingers superfamily protein) and *HOMEOBOX12* (*HB-12*) (Fig. 1d; Fig. S2; Supplementary Data Set S3). Among these early cytokinin-induced genes, we focused our studies on an ERF transcription factor *TINY2* (*ERF41*), whose node was closely associated with *LBD3*/*ASL9* (Fig. 1d). Analysis of fluorescent reporter lines revealed that *TINY2* is expressed in lateral meristems both in vascular cambium and periderm (Fig. 1f), consistent with single cell RNA sequencing data of the root secondary tissue (Lyu et al. 2025). At 7 days old, the *erf41/tiny2* mutant showed reduced vascular diameter and secondary xylem vessel number compared with Col-0 wild type (Fig. 1g; Fig. S3). Consistent with a role specifically in the activation stage, the *erf41/tiny2* vascular diameter and secondary xylem vessel number were similar to wild type at 14 days old (Fig. 1g; Fig. S3). Altogether this indicates that *TINY2* is a rapidly cytokinin-induced, cambium-expressed regulator of secondary growth activation.

At the late time point (T3, 8 to 24 h), the nodes with the largest number of edges were representing *AT1G47655* (*DNA-BINDING WITH ONE FINGER* (*DOF*) TF *DOF1.6*), *SVP*, *WLIM1*, *PHYTOCHROME-ASSOCIATED PROTEIN2* (*PAP2*), *ETHYLENE AND SALT INDUCIBLE3* (*ESE3*), *ETHYLENE-RESPONSIVE ELEMENT BINDING PROTEIN* (*EBP*)*/ERF072*, *ANT*, and *NAC099* (Fig. 1d; Fig. S2). We examined the DOF1.6 expression with a translational reporter line, *pDOF1.6:DOF1.6-YFP*, and observed a signal in the procambium and pericycle cells in young (5 d) roots with broader cambial expression in older (14 d) roots (Fig. 1f), consistent with single-cell RNA sequencing data of the root secondary tissue (Lyu et al. 2025). The *DOF1.6* homolog *DOF3.4* (Larrieu et al. 2022) exhibited a similar expression pattern in cambium and pericycle, whereas the reporter line of another close DOF1.6 homolog *DOF5.8* lacked prominent expression during root secondary growth (Fig. 1f). Inducible *DOF1.6* overexpression (*35S:XVE>>DOF1.6*) resulted in a markedly reduced secondary growth and primary root length (Fig. S4). Increased secondary xylem vessel number was observed in the 14-d-old *dof1.6* mutant (Fig. 1g; Fig. S3) which together with the lack of primary xylem axis differentiation in the induced (*35S:XVE>>DOF1.6*) line (Fig. S4) suggests that DOF1.6 is a cytokinin-inducible negative regulator of xylem development. The edges of *DOF1.6* were largely overlapping with those of *SVP* and *WLIM1* suggesting redundancy in the cambial GRN (Fig. 1d).

### Cytokinin-inducible TDIF-PXY signaling pathway promotes activation of secondary growth

**Figure 2:**
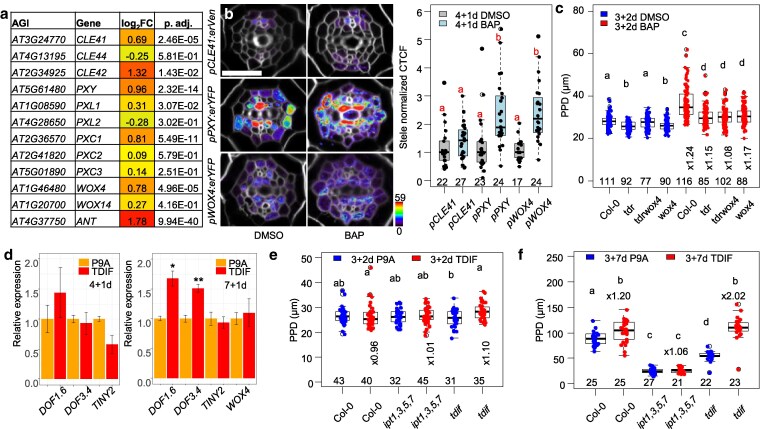
Cytokinin-inducible TDIF-signaling pathway regulates secondary growth activation. **a)** Transcriptional cytokinin response (4-h BAP, 1 µM) of genes involved in the TDIF-PXY signaling pathway in the *ipt1,3,5,7* mutant RNA-Seq data. **b)** Cross-sections of CLE41, PXY, and WOX4 marker lines after 4 + 1d DMSO and BAP (1 µM) treatments. Scale bar depicts 20 µm. Color key indicates fluorescence signal scale. Corrected total cell fluorescence (CTCF) was measured in Fiji and normalized to the median values of DMSO treatments. The experiment was repeated 3 times. **c)** Protophloem distances of Col-0, *pxy/tdr*, *tdrwox4* and *wox4* mutants after 3 + 2 days of DMSO and BAP (1 µM) treatments. The experiment was repeated 3 times. **d)** qPCR analysis of TDIF-peptide response after 4 + 1-d and 7 + 1-d treatments. Error bars mark ± SE, n = 3. Asterisks represent *P*-value from Welch 2-tailed *t*-test (*P*-value < 0.05 (*) and *P*-value < 0.01 (**). **e-f)** 3-d-old Col-0, *tdif* and *ipt1,3,5,7* plants were transferred to either mock (10 µM P9A) or TDIF (10 µM) plates for 2 (e) or 7 d (f). Protophloem distances from histological cross sections were quantified, the experiment was repeated 3 to 5 times. In b, c, e, f numbers depict number of roots analyzed with Fiji (n), the boxes in the box-and-whisker plots show the median and interquartile range, the whiskers indicate 1.5 × the interquartile range, and outliers are shown as circles. Individual data points are plotted on top of the box-and-whisker plots. Letters indicate statistical significance following Wilcoxon rank sum test with BH correction (adjusted *P*-value < 0.05). In c, e, and f fold changes calculated to the respective control median values are reported with “x”.

Due to the presence of *PXY* in the inferred cambial GRN and the reduced secondary growth of *pxy* (*pxy-5/tdif receptor* (*tdr*)) loss-of function mutant at 7 d (Fig. 1d, e), we hypothesized that cytokinin-induced cambium activation may at least partially be mediated by TDIF-PXY signaling. Accordingly, expression of TDIF-PXY signaling pathway members, such as TDIF-encoding *CLAVATA3/EMBRYO-SURROUNDING REGION-RELATED 41* (*CLE41*) and *CLE42* genes, a gene encoding PXY homolog *PXY-LIKE1* (*PXL1*) (Etchells et al. 2013), and *WOX4* and *ANT*, downstream factors of TDIF-PXY (Hirakawa et al. 2010; Eswaran et al. 2024), were increased from 4-h BAP treatment onwards (Fig. 2a; Supplementary Data Set S2). Similarly, expression of *PXY-CORRELATED1* (*PXC1*) (Wang et al. 2013) was also increased (Fig. 2a, Supplementary Data Set S2). Therefore, we analyzed the spatial cytokinin responsiveness of TDIF-PXY pathway with transcriptional reporter lines *pCLE41:erVenus*, *pPXY:erYFP*, and *pWOX4:erYFP*. Increased reporter expression after BAP treatment (4 + 1 d and 7 + 1 d) was observed in their endogenous expression domains: in phloem, pericycle (*pCLE41:erVenus*), cambium, and undifferentiated xylem cells (*pPXY:erYFP* and *pWOX4:erYFP*) (Fig. 2b; Fig. S5). Next, we studied the genetic interaction between cytokinin and TDIF-PXY in cambium activation. Cytokinin-induced cambium activation was reduced in the *pxy/tdr*, *tdrwox4,* and *wox4* mutants (Fig. 2c), indicating that a part of cytokinin-promoted cambium activation operates through TDIF-PXY pathway. Notably, *ASL9/LBD3*, *DOF1.6*, and *SVP* were the only genes to have edges with *PXY* in the cambial GRN (Fig. 1d, Supplementary Data Set S3), and only *DOF1.6* and *SVP* shared edges with *CLE41*. Next, to study whether the genes representing key nodes are transcriptionally TDIF regulated, we performed quantitative PCR analysis for *TINY2*, *DOF1.6*, and its homolog *DOF3.4*. *DOF1.6* and *DOF3.4* were significantly induced by 24-h TDIF treatment (7-d-old seedlings), and no significant change in expression of *TINY2* or *WOX4* upon TDIF treatment was observed (Fig. 2d). In contrast, a previous study reported that TDIF significantly induced WOX4 expression in young shoot tissue (Hirakawa et al. 2010), suggesting that the root cambium may have a lower sensitivity to TDIF-induced WOX4 expression than the shoot cambium or leaf vasculature. Overall, these findings support an idea of partial convergence of cytokinin and TDIF-PXY signaling pathways.

Subsequently, we tested whether TDIF treatment can prematurely activate cambium in wild-type, *ipt1,3,5,7*, and the quadruple mutant lacking TDIF peptide encoding CLE genes: *cle41 cle42 cle43 cle44* (hereafter *tdif*) (Smit et al. 2020). TDIF treatment (3 + 2 d 10 µM TDIF) increased the vascular diameter of *tdif* (Fig. 2e), and a longer TDIF treatment (3 + 7 d 10 µM TDIF) caused massive cell proliferation in Col-0 and *tdif* but not in the *ipt1,3,5,7* mutant (Fig. 2f; Fig. S6). Taken together, since TDIF treatment is unable to rescue *ipt1,3,5,7*, our data show that cytokinins regulate also TDIF-PXY–independent pathway(s) to promote early secondary growth.

### Cytokinin elicits auxin signaling and together activate secondary growth in an additive manner

**Figure 3:**
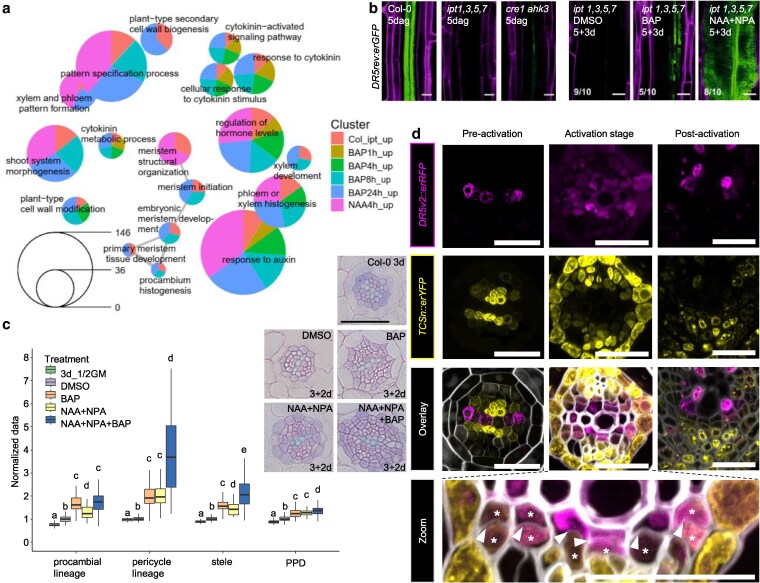
Auxin and cytokinin interact positively in the regulation of vascular cambium activity. **a)** Gene ontology enrichment analysis of biological processes among DEGs upregulated in comparison to the *ipt1,3,5,7* mutant DMSO control. Selected biological processes from 7-d-old plants are shown, see Supplementary Data Set S2 for complete over-representation analysis (adj. *P*-value < 0.05). **b)** Longitudinal view of *DR5rev:erGFP* roots 5 d after germination in Col-0, *ipt1,3,5,7*, and *cre1 ahk3* (left panel). *DR5rev:erGFP ipt1,3,5,7* grown for 5 d on ½ GM and transferred for 3 d onto DMSO (control), 5 µM BAP, or 5 µM NAA plus 20 µM NPA medium (right panel). Numbers of observed phenotypes are shown. Scale bar = 25 µm. **c)** 3-d-old Col-0 roots grown on ½ GM were either harvested or treated for 2 d with mock (DMSO), auxin (1 µM NAA + 20 µM NPA), cytokinin (1 µM BAP), or combined auxin and cytokinin (1 µM NAA + 20 µM NPA + 1 µM BAP). Cell file numbers and PPD were quantified with Fiji from histological cross sections, and data normalized to the median of treatment “DMSO”. Letters depict statistical analysis with Wilcoxon rank sum test (BH-adjusted *P*-value < 0.05). The boxes in the box-and-whisker plots show the median and interquartile range, the whiskers indicate 1.5 × the interquartile range. Outliers are not shown. Experiment was repeated 3 to 4 times, N(roots) = 41 to 86. Scale bar = 50 µm. **d)** Expression of auxin (*DR5v2:erRFP*) and cytokinin (*TCSn:erYFP*) signaling markers in root tip (pre-activation), 4- to 5-d-old roots (activation stage), and in 10-d-old roots (post-activation). In the zoomed image: asterix in (pro)cambium show overlap of expression. Arrowheads point to recent (pro)cambium divisions. Zoom × 3. Scale bars 20 µm.

Next, we used gene ontology (GO) enrichment analysis to elucidate the biological processes during cambium activation in the cytokinin time-course gene expression data. As the starting point, the genes downregulated in the *ipt1,3,5,7* mutant in comparison to the wild type were significantly enriched (adjusted *P*-value <0.05) for 99 GO categories, including “response to cytokinin”, “response to auxin”, “phloem or xylem histogenesis”, and “procambium histogenesis” (Fig. 3a; Supplementary Data Set S2). The majority of these categories (78/99, 79%) were also enriched in the cytokinin-induced time course data sets, indicative of the mutant phenotype rescue (Fig. 3a; Supplementary Data Set S2). Alongside “response to auxin”, processes such as “plant cell wall loosening” and “pattern specification process” were found enriched among genes upregulated in BAP and NAA treatment data sets (Fig. 3a). PIN-FORMED (PIN) proteins, which mediate polar auxin transport, are particularly interesting among “response to auxin” because PIN1, PIN3, and PIN7 are regulated by cytokinin to control auxin distribution during primary vascular tissue patterning in the root apical meristem (Bishopp et al. 2011). In contrast, during cambium activation, we found that *PIN5* and *PIN6* are induced by cytokinin (Supplementary Data Set S2).

Supporting the role of “phloem or xylem histogenesis” in our experimental setup, histological analysis showed that the *ipt1,3,5,7* mutant frequently has undifferentiated metaxylem, and this phenotype was rescued during the cytokinin time-course and interestingly also by 24-h NAA (Fig. S1). Since “response to auxin” was one of the major GO categories in our dataset, we studied auxin response in mutants with reduced cytokinin signaling. Auxin signaling marker *DR5rev:erGFP* was weakly expressed in the vasculature of both *ipt1,3,5,7* and cytokinin receptor mutant *cytokinin response1-12 arabidopsis histidine kinase3-3* (*cre1-12 ahk3-3*) (Fig. 3b). Furthermore, besides auxin, cytokinin treatment was also sufficient to induce the *DR5rev:erGFP* signal in *ipt1,3,5,7* (Fig. 3b). These data indicate that cytokinins positively regulate auxin signaling during cambium activation. To study the cytokinin-auxin interaction, we treated 3-d-old Col-0 roots with cytokinin, auxin and their combination. After 2 days, cytokinin and auxin treatments alone increased cell numbers in procambial and pericycle lineages in Col-0 roots, while the combined treatment resulted in the strongest secondary growth response (Fig. 3c). In this assay, auxin polar transport inhibitor 1-N-Naphthylphthalamic acid (NPA) was used to prevent NAA redistribution by auxin transporters, as we did previously (Mähönen et al. 2014). Similarly, without NPA, expression of cambial markers *pWOX4:erYFP* and *pANT:erRFP* as well as plasma membrane-localized periderm reporter *pPEROXIDASE15:mCherry-SYP122* (*pPER15:mCherry*) (Molina et al. 2024) were induced by cytokinin (BAP) treatment, but even stronger induction was observed after the combined NAA + BAP treatment in the wild-type background (Fig. S7). Auxin treatment alone did not significantly affect the expression of *pANT:erRFP*, *pWOX4:erYFP* or the cytokinin signaling reporter *TCSn:erYFP.* On the contrary, it induced *PER15* marker expression in the pericycle around emerging lateral roots (Fig. S7). Auxin signaling (*DR5v2:erRFP*) was increased by BAP treatment in xylem-adjacent cambial stem cells (Fig. S7), similar to the results obtained in *ipt1,3,5,7* background (Fig. 3a, b). Again, without NPA, auxin and cytokinin increased vascular diameter independently but most strongly together (Fig. S7). Auxin treatment (NAA + NPA) of the *ipt1,3,5,7* mutant resulted in increased vascular diameter, but this was likely a secondary effect due to extensive lateral root formation (Fig. S8). In conclusion, auxin and cytokinin have an additive effect on vascular cambium and pericycle activation.

### Cytokinin and auxin transiently occupy overlapping expression domains during vascular cambium activation

Next, we wanted to identify the endogenous auxin and cytokinin signaling domains during cambium activation. In the primary vasculature, within RAM, auxin and cytokinin signaling occupy mutually antagonistic domains in xylem axis and procambium, respectively (Fig. 3d, pre-activation) (Bishopp et al. 2011). Interestingly, our analysis showed that in 4- to 5-d-old roots, auxin and cytokinin signaling, as reported by *DR5v2:erRFP* and *TCSn:erYFP*, respectively, tends to transiently overlap prior and during cambium activation (Fig. 3d, activation stage). After the first procambium division, both daughter cells often exhibit transient expression of both auxin and cytokinin reporter. Gradually, high auxin signaling is confined to the xylem-side daughter cell, as we have previously reported (Smetana et al. 2019; Mäkilä et al. 2023), while the cytokinin reporter becomes restricted to the phloem-side daughter cell. After subsequent cell division rounds, auxin and cytokinin signaling maxima become prominent in opposing domains (Fig. 3d, post-activation). Similar results were obtained with a combination of *DR5rev:erGFP* and *pARR5:erRFP* (for auxin and cytokinin signaling, respectively) whose expression transiently overlapped in procambial cells of 5- and 7-d-old Col-0 roots (Fig. S8). While cell-to-cell signaling is the typical mechanism for cytokinin-auxin crosstalk in tissue patterning, our data suggests that a brief overlap of these 2 hormones may also act as a trigger for developmental transitions, such as during cambium activation.

### Auxin signaling is required for cytokinin-induced cambium activation

**Figure 4:**
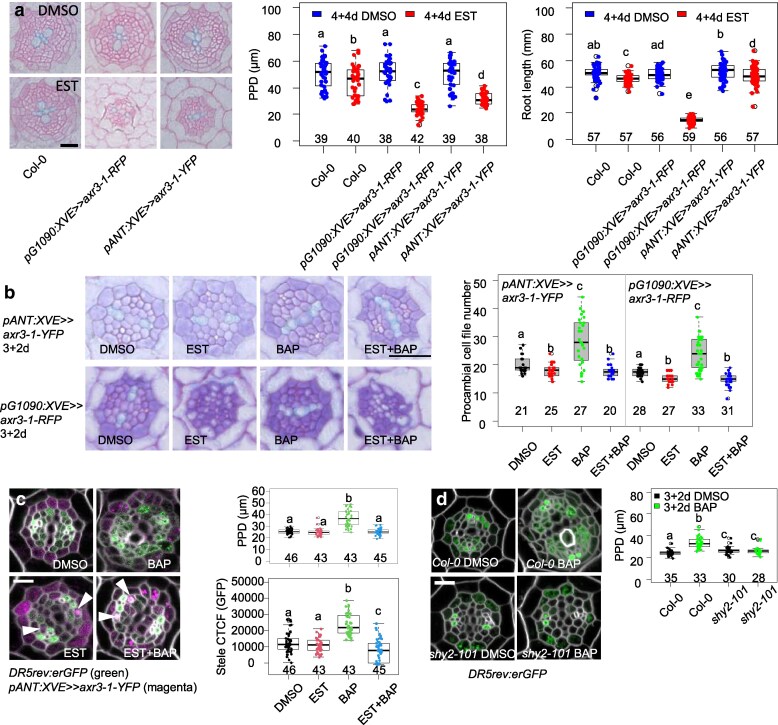
Auxin signaling is required for cytokinin-induced cambium activation. **a)** Col-0 and estradiol-inducible lines targeting auxin signaling (*pG1090:XVE>>axr3-1-RFP*, *pANT:XVE>>axr3-1-YFP*) were treated with DMSO or 5 μm EST 4 + 4 d. Protophloem distances (PPDs) and root lengths were measured in Fiji. Scale bar = 20 μm. The experiment was repeated 4 times. **b)**  *pG1090:XVE>>axr3-1-RFP* and *pANT:XVE>>axr3-1-YFP* were treated with DMSO, 1 μm BAP, 5 μm EST, and EST + BAP 3 + 2 days. Procambial cell file numbers were quantified from plastic sections. Scale bar = 25 μm. The experiment was repeated 3 times. **c)**  *DR5rev:erGFP pANT:XVE>>axr3-1-YFP* roots were treated with DMSO, 1 μm BAP, 5 μm EST, and EST + BAP 3 + 2 d. Arrowheads indicate nuclear axr3-1-YFP signal. PPD and GFP CTCF from the stele were quantified in Fiji. The experiment was repeated 3 times. Scale bar = 10 μm. **d)**  *DR5rev:erGFP* and *DR5rev:erGFP shy2-101* were treated with DMSO and 1 μm BAP 3 + 2 d. PPD was quantified in Fiji. The experiment was repeated 4 times. Scale bar = 10 μm. In a, b, c, and d numbers depict number of roots analyzed with Fiji (n). The boxes in the box-and-whisker plots show the median and interquartile range, the whiskers indicate 1.5 × the interquartile range, and outliers are shown as circles. Individual data points are plotted on top of the box-and-whisker plots. Letters indicate statistical significance following Wilcoxon rank sum test with BH correction (adjusted *P*-value < 0.05).

Ubiquitous inducible overexpression of *auxin resistant3-1* (*axr3-1*), a dominant negative regulator of auxin signaling, affects both primary (Bishopp et al. 2011; Mähönen et al. 2014; Siligato et al. 2016) and secondary development (Smetana et al. 2019) of root vascular tissues. To study auxin signaling specifically during early secondary growth, we expressed *axr3-1-YFP* under estradiol (EST)-inducible cambial *pANT:XVE* construct in 4-d-old plants, which resulted in almost complete inhibition of early secondary growth without simultaneous adverse effects on root primary growth seen with ubiquitous *pG1090:XVE >>axr3-1-RFP* construct (Fig. 4a). To study the auxin-cytokinin interaction during cambium activation, we treated 3-d-old seedlings with cytokinin with and without axr3-1 induction. Procambial cell file number was statistically significantly reduced by axr3-1 induction in *pANT:XVE>>axr3-1-YFP* and *pG1090:XVE >>axr3-1-RFP* lines in both EST and EST + BAP treatments, whereas BAP treatment alone caused increased procambial cell file number (Fig. 4b). We also analyzed auxin signaling marker *DR5rev:erGFP* in the *pANT:XVE>>axr3-1-YFP* background. Cytokinin treatment induced *DR5rev:erGFP* expression in the procambium and increased vascular diameter (protophloem distance [PPD]) (Fig. 4c). These effects were absent when plants were simultaneously treated with EST to induce *axr3-1-YFP*, and *DR5rev:erGFP* fluorescence was further reduced under these conditions (Fig. 4c). Lack of increased PPD by 2d cytokinin treatment was also observed in the dominant negative *short hypocotyl2-101* (*shy2-101*) mutant (Fig. 4d). However, SHY2 is a cytokinin-inducible negative regulator of auxin signaling (Ioio et al. 2008), and therefore we estimate that cytokinin treatment makes *shy2-101* mutant phenotypes more severe. Altogether, these results indicate that auxin signaling is required for cytokinin-induced cambium activation. During prolonged (7 d) treatment of *pANT:XVE>>axr3-1-YFP* plants with EST + BAP, a partial rescue of the vascular diameter was observed (Fig. S9); this suggests that cytokinin signaling may act also independently from auxin after activation of secondary growth.

### Transcriptomic analysis reveals both overlapping and independent DEGs downstream of auxin and cytokinin signaling

**Figure 5:**
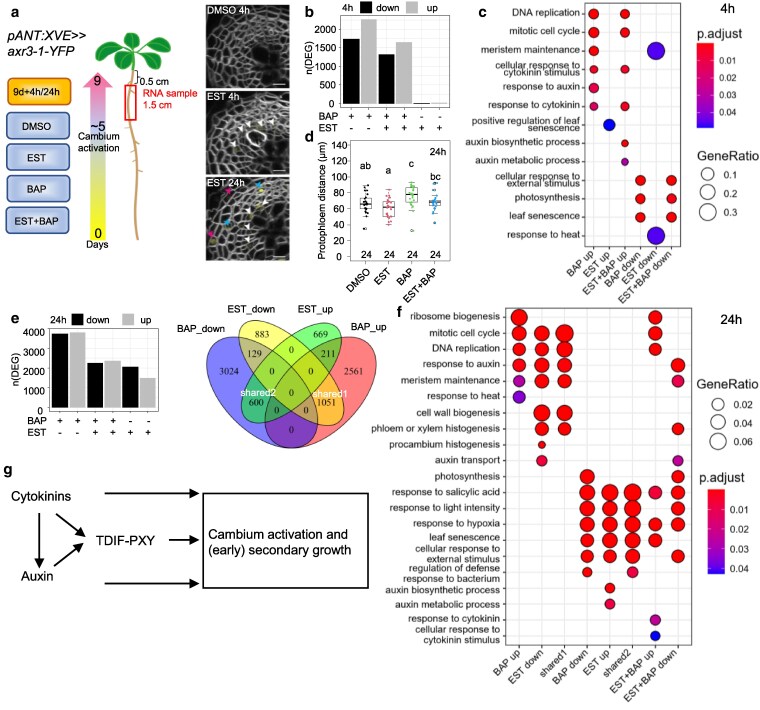
Transciptomic profiling of auxin-cytokinin interaction in secondary growth regulation. **a)** Scheme for harvesting root sections for RNA-Seq. Cross sections of *pANT:XVE>>axr3-1-YFP* roots after 4- and 24-h EST treatment. White arrowheads point to nuclear YFP in cambium, blue in phloem, and pink in periderm. Scale bars 10 µm. **b)** Number of differentially expressed genes (DEGs) (abs(log_2_FC) ≥ 0.5, qval ≤ 0.05) after 4-h treatments. **c)** Selected GO enrichment categories in 4-h DEGs. **d)** Secondary growth phenotypes of the 24-h samples. Protophloem distances were quantified from cross sections, letters indicate statistical significance following *t* test (BH-adjusted *P*-value < 0.05). The experiment was repeated 3 times. Numbers depict number of roots analyzed with Fiji (n). The boxes in the box-and-whisker plots show the median and interquartile range, the whiskers indicate 1.5 × the interquartile range, and outliers are shown as circles. Individual data points are plotted on top of the box-and-whisker plots. **e)** Number of differentially expressed genes (abs(log_2_FC) ≥ 0.5, qval ≤ 0.05) after 24-h treatments in comparison to the DMSO control (barplot) and the overlap of selected gene lists as Venn diagram. **f)** Selected GO enrichment categories in 24-h DEGs. **g)** Model of converging hormone and peptide signaling pathways in secondary growth regulation. In c and f, the GeneRatio size depicts the ratio of genes assigned to each biological process per DEG list analyzed gene number, the color scale of p.adjust depicts the adjusted *P*-value significance.

Next, we used the estradiol-inducible *pANT:XVE>>axr3-1-YFP* line to profile the transcriptomic interaction of auxin and cytokinin signaling in cambium during early secondary growth. Samples from roots with already active cambium treated with estradiol (EST), cytokinin (BAP), or both were harvested with the DMSO-treated controls at 4- and 24-h time points for RNA-Seq (Fig. 5a). Cambial axr3-1-YFP signal was present after 4-h EST treatment (Fig. 5a), but few genes were yet differentially expressed by EST treatment alone (Fig. 5b; Supplementary Data Set S5). However, 4-h EST + BAP treatment resulted in reduced number of differentially expressed genes (DEGs) in comparison to 4-h BAP-treatment (log_2_FC ≥ 0.5, qval ≤ 0.05; Fig. 5b; Supplementary Data Set S5). Markedly, biological process “response to auxin” was not enriched in 4-h EST + BAP sample DEGs, unlike in 4-h BAP alone (Fig. 5c; Supplementary Data Set S5). Altogether this suggests that cambial auxin signaling is required for proper transcriptional cytokinin response; that is, this finding further supports the idea that auxin acts at least partially downstream of cytokinin during early secondary growth.

After 24-h treatment, increased vascular diameter in BAP-treated plants was present (Fig. 5d). Combined EST + BAP treatment caused no statistically significant change in secondary growth, and the number of DEGs was reduced in comparison to the BAP treatment (Fig. 5d, e). Twenty-four–hour EST treatment led to *axr3-1-YFP* induction in cambium and in some phloem and periderm cells (Fig. 5a) and to the downregulation of 2,063 genes, of which the majority (51%, 1,051 genes) were positively transcriptionally regulated by 24-h BAP treatment (Fig. 5e). Correspondingly, processes linked to cell division (mitotic cell cycle, DNA replication) were enriched among genes downregulated in the EST-treated plants, upregulated in the BAP-treated plants and among the genes regulated (although in opposite directions) by both treatments (list “shared1”, 1,051 genes) (Fig. 5f). Therefore, cambial cell cycle represents a key process positively controlled by auxin and cytokinin signaling pathways. Response to auxin, auxin transport, and developmental processes such as phloem/xylem histogenesis and procambium histogenesis were also enriched among genes downregulated after 24 h EST treatment (Fig. 5f; Supplementary Data Set S5). Because procambium histogenesis includes genes belonging to the TDIF-PXY signaling pathway, this result is in line with our earlier finding that auxin signaling promotes PXY expression (Smetana et al. 2019). The genes upregulated by EST (axr3-1 induction) were enriched for biotic and abiotic stress responses; these processes were enriched also among genes downregulated by BAP treatment as well as among the shared genes regulated by both individual treatments (list “shared2”, 600 genes) (Fig. 5f; Supplementary Data Set S5). Next, we wanted to test whether auxin and cytokinin are continuously required for secondary growth after its activation. Long-term EST treatment of *pANT:XVE>>axr3-1-YFP* plants induced when 8 d old (after cambium activation) ceased root radial growth and also increased cell size (Fig. S10). We also treated *ipt1,3,5,7* plants with cytokinin either for 2d only to achieve cambium activation or continuously for 9 d (Fig. S11). Roots with only 2-d treatment ceased to grow radially after the initial cambial cell divisions. Altogether, these data show that auxin and cytokinin are continuously required for secondary growth driven by vascular cambium. In conclusion, our data suggests that auxin and cytokinin regulate large sets of genes, both independently as well as in highly cooperative manner, associated with several developmental processes during activation and early stages of secondary growth (Fig. 5g).

## Discussion

### Auxin and cytokinin signaling interaction in cambium establishment

Auxin and cytokinin signaling and homeostasis interact in many ways, most of which have been characterized as antagonistic (Ioio et al. 2008; Bishopp et al. 2011). Here, we discovered that cytokinin positively regulates auxin signaling to promote the onset of secondary development. First, we used the *ipt* quadruple mutant as a tool to synchronously activate the vascular cambium in the Arabidopsis root with cytokinin treatment. Auxin signaling as a biological process was enriched among cytokinin-upregulated genes in the *ipt1,3,5,7* mutant during several time points (Fig. 3a), and the auxin signaling marker *DR5v2:erRFP* expression increased in the xylem-adjacent cambial stem cells of young Col-0 roots after BAP treatment (Fig. S7). Moreover, we find that in Arabidopsis auxin signaling is downregulated in the stele of both cytokinin biosynthesis (*ipt1,3,5,7*) and signaling (*cre1 ahk3*) mutants, indicating that proper auxin signaling requires cytokinin signaling. Similar results have been obtained from *Populus* trees overexpressing cytokinin biosynthesis gene *IPT7* as they showed higher levels of both auxin and transcriptional auxin responses (Immanen et al. 2016). Our experiments where we combined cytokinin treatment and cambium-inducible *axr3-1,* showed auxin signaling being part of fast transcriptional cytokinin responses. However, long-term experiments suggested that auxin and cytokinin signaling act also in parallel. This was supported by simultaneous cytokinin and auxin treatments additively resulting in the vascular cambium and pericycle activation more efficiently than either hormone alone. Our marker analysis showed that auxin and cytokinin signaling transiently overlap prior and during the first periclinal cell divisions, soon after which auxin signaling is restricted into the inner organizer cell/xylem domain (Smetana et al. 2019) and cytokinin into the outer daughter/stem cell. Similar separation of initially overlapping cytokinin and auxin signaling domains can be found during de novo meristem formation in tomato (*Solanum lycopersicum*) adventitious root system (Omary et al. 2022 ). Therefore, we postulate that cambium stem cell establishment may follow a common mechanism in which both auxin and cytokinin are required for meristem formation but as it becomes functional, the signaling domains are mostly separated.

### Cytokinin as a major activator of cambial cell divisions

Cytokinin-induced gene expression is dependent on type B response regulators ARR1, ARR2, and ARR10, which bind to target sequences more strongly upon cytokinin treatment (Zubo et al. 2017; Xie et al. 2018). Moreover, ARR10 binding peaks overlap with differentially accessible chromatin regions (DARs) in response to cytokinin (Potter et al. 2018), suggesting that type B response regulators act as pioneer transcription factors increasing chromatin accessibility to other transcription factors. In this study, we treated the cytokinin biosynthesis mutant *ipt1,3,5,7* with BAP, NAA, and TDIF peptide. Whereas BAP treatment was able to rescue the secondary growth phenotype, NAA and TDIF were not able to do so. This could suggest that while auxin and TDIF peptide signaling are required for early secondary growth, the cytokinin-regulated DARs are necessary for the secondary growth to fully progress. Interestingly, also auxin is known to regulate chromatin status through MP-dependent interactions with SWItch/Sucrose Non-Fermentable (SWI/SNF) chromatin remodeling ATPases (Wu et al. 2015), a mechanism with putative role also in the auxin-dependent cambium activation. This may be facilitated by several cambial ARFs, as the amiRNA line targeting MP in *arf7,19* background is able to activate cambium but lacks secondary xylem vessels (Smetana et al. 2019). Future studies could elucidate the overlap of the chromatin status changes upon cytokinin, auxin and their combined treatment, and whether this could explain the additive effect of these 2 plant hormones on secondary growth regulation. Furthermore, the upstream mechanisms establishing the cambial cytokinin and auxin signaling domains remain yet unelucidated.

In the active vascular cambium, we found transcriptional auxin and cytokinin responses to include distinct but also largely overlapping target genes. For instance, transcripts assigned to cell divisions were positively regulated by both hormones. Cytokinin has been directly linked to mitotic cell divisions also through subcellular translocation of MYB TFs (Yang et al. 2021). Cytokinin and auxin transcriptionally downregulated genes involved in stress responses, suggesting a negative relationship between stress and cambium activity. However, some stress-responsive genes, such as ERFs, have been shown to be required for secondary growth (Hoang et al. 2020). Recently LBD11 was shown to mediate oxidative stress signaling to promote cambium proliferation and induce also negative feed-back regulation of stress signaling (Dang et al. 2023). Therefore, the role of cambium-expressed stress-regulated genes needs to be assessed more in detail and also with spatio-temporal resolution.

### ERF and DOF transcription factors participate in the intertwined network of vascular cambium regulation

Previous studies have found interactions between hormonal and TDIF-PXY-signaling pathways (Suer et al. 2011; Han et al. 2018; Smetana et al. 2019; Smit et al. 2020). Additionally, TDIF-PXY-WOX signaling has been reported to act in parallel with AT-HOOK MOTIF CONTAINING NUCLEAR LOCALIZED 15 (AHL15)-dependent cytokinin production (Rahimi et al. 2022). In this study, we find TDIF-PXY signaling pathway downstream of both cytokinin and auxin signaling during early secondary growth (Fig. 5g). Moreover, several tiers of TDIF-PXY pathway expressed in phloem and cambial domains were induced by BAP, including *ANT* and *WOX4*. Phloem-localized cytokinin signaling regulates non–cell-autonomously cambial activity during secondary growth of *Populus* stems (Fu et al. 2021), and it is interesting to speculate a possible link to TDIF-PXY signaling. In the cambium activation GRN inferred in our study, TDIF-encoding *CLE41* and *PXY* both formed edges with *DOF1.6* and *SVP*. Interestingly, *DOF1.6* was transcriptionally induced by TDIF peptide, suggesting that *DOF1.6* is converging cytokinin and TDIF pathways. The *dof1.6* phenotype suggested a negative regulation of xylem differentiation. Cytokinin and TDIF peptide signaling both have inhibitory effect on primary and secondary xylem vessels, respectively, whereas auxin is known to promote secondary vessel formation (Smetana et al. 2019). The *axr3-1* induction reduced *DOF1.6* expression (Supplementary Data Set S5) suggesting a role for *DOF1.6* also during auxin signaling. DOF1.6 belongs to a transcriptional module coexpressed with leaf vasculature formation in the VISUAL system (Furuya et al. 2021) and is also expressed in the xylem side of inflorescence stem cambium (Shi et al. 2021). DOF1.6 homolog DOF3.4 has been implicated in cell cycle regulation (Skirycz et al. 2008), and here we find that *DOF3.4* is expressed in both cambia and also induced by TDIF treatment. Additionally, we identified *TINY2* (*ERF41*) from the cambial GRN having a role specifically in cambium activation (Fig. 1g). This may be characteristic for genes rapidly induced by cytokinin, such as for *LBD3* and *LBD4*, transcription factors required for cytokinin-induced cambium initiation (Ye et al. 2021). Therefore, the cambial GRN may help to elucidate additional transcription factors having a role during cambium activation.

In conclusion, we have shown that plant hormones auxin and cytokinin and TDIF-peptide signaling regulate early vascular cambium activity in a co-operative manner and that these signaling pathways also converge in a gene regulatory network inferred during cambium activation.

## Materials and methods

### Plant materials, growth conditions, and molecular cloning

The previously characterized Arabidopsis (*Arabidopsis thaliana*) wild-type and mutant lines used in this work were Col-0, *ipt1,3,5,7* (Miyawaki et al. 2006), *cre1-12 ahk3-3* (Higuchi et al. 2004), *shy2-101* (Goh et al. 2012), *tdif* (*cle41, 42, 43, 44*) (Smit et al. 2020), *svp-41* (Gregis et al. 2006)*, erf072-1, lbd4-1, lbd4-2*, and *asl9/lbd3* (Zhang et al. 2019), *pxy-5/tdr, wox4*, and *tdrwox4* (Hirakawa et al. 2010). T-DNA insertion lines for *tiny2* and *dof1.6* were ordered from the Nottingham Arabidopsis Stock Center (NASC) with codes SAIL_1211_D04 and SALK_016252C, respectively. The primers used for genotyping are listed in Supplementary Data Set S1. The previously published transgenic lines used in this work were *pCLE41:erVEN* (Eswaran et al. 2024)*, pPXY:erYFP* (Smetana et al. 2019), *pG1090:XVE>>axr3-1-RFP* (Mähönen et al. 2014), *pWOX4:erYFP* (Suer et al. 2011), *pANT:erRFP* and *DR5v2:erRFP* (Mäkilä et al. 2023), *TCSn:erYFP* (Ye et al. 2021), *DR5rev:erGFP* (Friml et al. 2003), *pPER15:mCherry-SYP122* (Molina et al. 2024), and *pARR5:erRFP* (Siligato et al. 2016).

Plants were grown on ½ growth media (1/2 GM) containing 0.8% Plant Agar (Duchefa), 1% sucrose (Duchefa), 0.5× Murashige Skoog salts including vitamins (Duchefa) and pH adjusted to 5.7 to 5.8 with 20× MES buffer (Mes monohydrate, Duchefa). The plates were placed vertically in a 23 °C growth chamber with long-day settings (16 h light and 8 h dark).

Multisite Gateway technology was used in the molecular cloning of the following constructs: *pDOF1.6:DOF1.6-YFP*, *pDOF3.4:erRFP*, *pDOF5.8:YFP*, *pTINY2:erRFP*, *35S:XVE>>DOF1.6*, and *pANT:XVE>>axr3-1-YFP.* Wild-type Col-0 plants were transformed with the floral dip method and transgenic lines used for experiments were screened to be representatives of single insertion homozygous lines. Primers and recombinant DNAs used are listed in the Supplementary Data Set S1.

### Microarray hybridizations and *ipt1,3,5,7* RNA-Seq

Plant material for microarray hybridizations (7-d-old Col-0 and *ipt1,3,5,7* plants) were grown, treated, harvested, and RNA extracted as described in (Randall et al. 2015). The 14-d-old Col-0 roots were grown independently vertically on ½ GM plates prior to harvesting the main root samples aged between 7 and 14 days. The extracted RNAs were processed and hybridized to Agilent 4 × 44 K slides as in (Furuta et al. 2014). The experiment was repeated in triplicate. Log_2_-fold changes and false discovery rate corrected *P*-values were calculated for each probe with scripts in R (Supplementary Data Set S2). Genewise log_2_-fold changes were calculated by averaging gene-specific probe values. Additionally, biological replicates of *ipt1,3,5,7* mutant control (1 h DMSO) and 4-h BAP treatment were also analyzed with RNA-Seq. Library preparation and transcriptome sequencing were performed at BGI (https://www.bgi.com/global). The RNA-Seq data quality analysis and trimming of the reads was performed as previously described in (Xu et al. 2015). The mapping was performed with Bowtie2, the counting of reads per gene was done using HTSeq and differential gene expression analyzed with Deseq2 package in Chipster (Kallio et al. 2011). The log_2_ fold changes and adjusted *P*-values are available in Supplementary Data Set S2.

### Network analysis

Gene regulatory network (GRN) inference for cambium activation was carried out by following the GENIST method (De Luis Balaguer et al. 2017). Briefly, first, differentially expressed genes (probe-wise microarray data) from 2 consecutive time points were selected using comparison-specific cutoff values. Next, the T1 (BAP1h-BAP4h), T2 (BAP4h-BAP8h), and T3 (BAP8h-BAP24h) probe lists were each clustered using their gene expression values of the *ipt1,3,5,7* DMSO 1 h, Col-0 DMSO1h, Col-0 old ,and *ipt1,3,5,7* NAA4h samples. From T1, T2, and T3 the clusters containing ANT were selected for GRN. Finally, the GRN depicting the ANT cluster at all time points was inferred. Data used for network inference is available in Supplementary Data Set S3.

### Histological analysis of cross sections

Historesin-embedded roots were processed for histological analysis as described in (Idänheimo et al. 2014). Root vasculature parameters 2 to 5 mm below root-hypocotyl junction were analyzed from microscope images with Fiji. From each biological repeat used for microarray hybridizations, 10 plants per 7-d-old samples were analyzed for their protophloem distance and cell numbers. Statistical analysis was performed with scripts in R and the results are summarized in Supplementary Data Set S4.

### Confocal analysis

Using a protocol modified from (Smetana et al. 2019), samples were fixed with 4% paraformaldehyde solution (PGA, Sigma) in 1 × phosphate-buffered saline (PBS). After fixation samples were washed with PBS and embedded in 4% agarose. Vibratome was used to cut embedded samples into 200-µm-thick sections, 50-µm sections were used for root tip images. Cell walls were stained with SR2200 in PBS (1:1,000, Renaissance Chemicals) and confocal analysis was performed with Leica Stellaris 8 confocal microscope (63 × objective). In longitudinal confocal images, cell walls were stained with propidium iodide and imaged with Leica TCS SP5II HCS A. Sections were imaged in either PBS or water. All confocal images with multiple channels were images in sequential scanning mode. Confocal settings may have varied between experiments but always stayed the same for the experimental sample and respective control. To optimize the cell wall staining, the signal was adjusted when necessary, during imaging and may thus vary between the sample and respective control.

### Fluorescence quantifications

Corrected total cell fluorescence (CTCF) from confocal pictures was measured in Fiji, by selecting the area expressing marker gene or the stele as the region of interest (ROI). Integrated density ie sum of pixel values in selected areas divided by the area, was then calculated for the ROI. Additionally, intensity of the background signal was measured from an area without marker expression. CTCF was then calculated by the following formula: CTCF = integrated density − (area of selected cell × mean fluorescence of background readings). For 3 + 2-d marker analysis, roots were laterally imaged on/through growth media with a stereofluorescence microscope (Leica M165 FC). Fluorescence profile from 10-pixel-wide line encompassing 2 mm in length per root was quantified with Fiji. Average fluorescence per root was normalized to the DMSO control average and used in the statistical analysis in R (Supplementary Data Set S4).

### pANT:XVE>>axr3-1-YFP RNA-Seq

Homozygous *pANT:XVE>>axr3-1-YFP* seeds were surface sterilized with bleach, 70% EtOH, washed with sterile water and stratified 2 to 3 days. Two rows of seeds (20 seeds per row) were plated in 0.1% sterile agarose on autoclaved Nitex mesh placed on the surface of each growth plate (0.8% Plant Agar (Duchefa), 0.5 × MS media with vitamins, 1% sucrose, pH adjusted with MES to 5.7). Plates sealed with micropore tape were put to grow vertically on a single transparent tray in the Sanyo controlled environment growth chambers. For treatment, 9-d-old plants were transferred on the Nitex mesh to plates supplemented with either 5 µM estradiol, 1 µM BAP, 5 µM estradiol together with 1 µM BAP or the DMSO control. After 24 h, 1.5-cm-long main root segments were harvested by cutting the roots 0.5 cm (start) and 2 cm below the root-hypocotyl junction and flash-frozen in liquid N. The experiment was repeated 3 times for each time point. Remaining roots attached to hypocotyls were transferred to fixation solution for plastic sections 0.5 cm below the root-hypocotyl junction to analyze the vascular phenotypes. The experiment was repeated similarly for the 4-h time point.

Total RNA was extracted with the GeneJet Plant RNA purification mini kit (Thermo Scientific). RNA concentrations were measured with NanoDrop 1000 Spectrophotometer and an aliquot was used for RNA quality analysis with Bioanalyzer 2100 (Agilent Technologies). DNase treatment with Heat&Run gDNA removal kit (ArcticZymes) and ribosomal RNA removal with Plant Ribo-Zero rRNA Removal Kit (Illumina) were done prior to the strand-specific library preparation with a TruSeq Stranded Total RNA Library prep kit (Illumina). Samples were sequenced with NextSeq 500 and the raw RNA-Seq reads were quality checked using fastqc. The adapters and barcodes were removed using Trimmomatic [v 0.36] (Bolger et al. 2014). To align, Kallisto [v.0.43.0] (Bray et al. 2016) was used, with 4,000 bootstrap replicates, to map the high quality reads to *Arabidopsis thaliana* Reference Transcript Dataset 2 (AtRTD2) (Zhang et al. 2017). The gene-wise raw count data was used for analyzing differential gene expression (abs(log_2_ fold change) ≥ 0.5, adjusted *P*-value ≤ 0.05) between each treatment and the respective DMSO control with edgeR in the Chipster platform (Kallio et al. 2011; Supplementary Data Set S5). Library preparation and RNA sequencing were carried out at the DNA Sequencing and Genomics Laboratory, Institute of Biotechnology, University of Helsinki.

### Hierarchical clustering, gene ontology enrichment, and gene list comparisons

Hierarchical clustering of selected genewise log_2_-fold changes was performed using the pheatmap package in R without scaling (Kolde 2010). Cutoffs for microarray and RNA-Seq DEGs (|log_2_FC|≥0.05, adjusted *P*-value ≤0.05) and subsequent GO enrichment analysis (adjusted *P*-value cutoff 0.05) was done in R with Clusterprofiler package (Wu et al. 2021). Comparisons of the cambium activation GRN to published datasets was done with the GeneOverlap package (Shen 2014) in R. Jaccard index and Fisher's exact test with *P*-value correction for multiple testing (Benjamin-Hochberg) were calculated using TAIR10 with 33,602 genes as the background.

### qPCR

Plants were transferred to 1/2GM plates containing either 10 μM TDIF peptide or P9A control peptide (nonfunctional, mutated TDIF peptide unable to bind to PXY) (Ito et al. 2006) to induce the expression of the target genes for 24 h. Two-centimeter root segments, below the hypocotyl/root junction, were harvested from the primary roots of 14 to 16 individual plants/treatment for RNA purification. The experiment was repeated 3 times for each treatment. From T-DNA lines grown on 1/2 GM plates, whole roots from 20 to 35 plants per sample were harvested.

Total RNA was isolated from root samples using the RNeasy Plant Mini Kit (QIAGEN) with an on-column DNase I treatment (QIAGEN). cDNA synthesis was performed using the iScript cDNA Synthesis Kit (Bio-Rad Laboratories) following the manufacturer's protocol. Quantitative reverse-transcription PCR (qRT-PCR) was conducted in a 10-μL reaction volume using the HOT FIREPol EvaGreen qPCR Mix Plus (Solis BioDyne) on a CFX384 Real-Time PCR System (Bio-Rad). PCR program was as follows: 95 °C for 5 min, 45 cycles (95 °C for 10 s, 59 °C for 10 s, 72 °C for 15 s), melting curve analysis.

Three technical repeats were included for each biological repeat during qRT-PCR. Detected expression levels were normalized using the Comparative CT Method (ΔΔCT method, (Livak and Schmittgen 2001)) using multiple reference genes (*18S*, *UBIQUITIN-CONJUGATING ENZYME21* (*UBC21*) and *ELONGATION FACTOR-1a* (*EF1a*)). Statistical analyses were performed using 2-tailed Welch *t* test (Supplementary Data Set S4). T-DNA line analysis was performed by normalizing detected expression levels to *UBC21* and *PROTEIN PHOSPHATASE 2A SUBUNIT A3* (*PP2AA3*). All primers used for qRT-PCR are listed in Supplementary Data Set S1.

### Accession numbers

The accession numbers of genes represented in the cambium activation GRN (Fig. 1d) are listed in the Supplementary Data Set S3. Additional accession numbers of Arabidopsis genes in this article are provided in the Supplementary Data Set S1.

## Supplementary Material

koag059_Supplementary_Data

## Data Availability

The transcriptomic data underlying this article are available in the NCBI Gene Expression Omnibus (GEO) with the accession number GSE293194 and also in the NCBI Bioproject database with the accession number PRJNA1243323.

## Dive Curated Terms

The following phenotypic, genotypic, and functional terms are of significance to the work described in this paper:

WOX4 Gramene: AT1G46480
WOX4 Araport: AT1G46480

CLE41 Gramene: AT3G24770
CLE41 Araport: AT3G24770

WLIM1 Gramene: AT1G10200
WLIM1 Araport: AT1G10200

ARR10 Gramene: AT4G31920
ARR10 Araport: AT4G31920

SHY2 Gramene: AT1G04240
SHY2 Araport: AT1G04240

LBD4 Gramene: AT1G31320
LBD4 Araport: AT1G31320

CYCD3;1 Gramene: AT4G34160
CYCD3;1 Araport: AT4G34160

VND6 Gramene: AT5G62380
VND6 Araport: AT5G62380

ANT Gramene: AT4G37750
ANT Araport: AT4G37750

IPT1 Gramene: AT1G68460
IPT1 Araport: AT1G68460

IPT3 Gramene: AT3G63110
IPT3 Araport: AT3G63110

IPT5 Gramene: AT5G19040
IPT5 Araport: AT5G19040

IPT7 Gramene: AT3G23630
IPT7 Araport: AT3G23630

LBD3 Gramene: AT1G16530
LBD3 Araport: AT1G16530

PXY Gramene: AT5G61480
PXY Araport: AT5G61480

DOF1.6 Gramene: AT1G47655
DOF1.6 Araport: AT1G47655

DOF3.4 Gramene: AT3G50410
DOF3.4 Araport: AT3G50410

DOF5.8 Gramene: AT5G66940
DOF5.8 Araport: AT5G66940

CLE44 Gramene: AT4G13195
CLE44 Araport: AT4G13195

CLE43 Gramene: AT1G25425
CLE43 Araport: AT1G25425

CLE42 Gramene: AT2G34925
CLE42 Araport: AT2G34925

AXR3 Gramene: AT1G04250
AXR3 Araport: AT1G04250

PER15 Gramene: AT2G18150
PER15 Araport: AT2G18150

PIN6 Gramene: AT1G77110
PIN6 Araport: AT1G77110

SVP Gramene: AT2G22540
SVP Araport: AT2G22540

ERF072 Gramene: AT3G16770
ERF072 Araport: AT3G16770

PIN5 Gramene: AT5G16530
PIN5 Araport: AT5G16530

PTL Gramene: AT5G03680
PTL Araport: AT5G03680

ATHB5 Gramene: AT5G65310
ATHB5 Araport: AT5G65310

IDD2 Gramene: AT3G50700
IDD2 Araport: AT3G50700

GIS3 Gramene: AT1G68360
GIS3 Araport: AT1G68360

HB-12 Gramene: AT3G61890
HB-12 Araport: AT3G61890

ESE3 Gramene: AT5G25190
ESE3 Araport: AT5G25190

NAC099 Gramene: AT5G56620
NAC099 Araport: AT5G56620

TINY2 Gramene: AT5G11590
TINY2 Araport: AT5G11590

PAP2 Gramene: AT4G29080
PAP2 Araport: AT4G29080

AT5G10970 Gramene: AT5G10970
AT5G10970 Araport: AT5G10970

AT5G57150 Gramene: AT5G57150
AT5G57150 Araport: AT5G57150

AT2G40200 Gramene: AT2G40200
AT2G40200 Araport: AT2G40200

PXL1 Gramene: AT1G08590
PXL1 Araport: AT1G08590

PXC1 Gramene: AT2G36570
PXC1 Araport: AT2G36570

CRE1 Gramene: AT2G01830
CRE1 Araport: AT2G01830

AHK3 Gramene: AT1G27320
AHK3 Araport: AT1G27320

ARR5 Gramene: AT3G48100
ARR5 Araport: AT3G48100
