# Supplementary material for: Plant hormone and peptide signaling converge in the genetic network regulating cambium activation in Arabidopsis roots
Source: Plant Cell. 2026 Mar 13;38(6):koag059. doi: 10.1093/plcell/koag059 (PMC13259593; doi:10.1093/plcell/koag059)
Supplement: koag059_Supplementary_Data [file koag059_supplementary_data.zip › Supplementary Data.pdf]

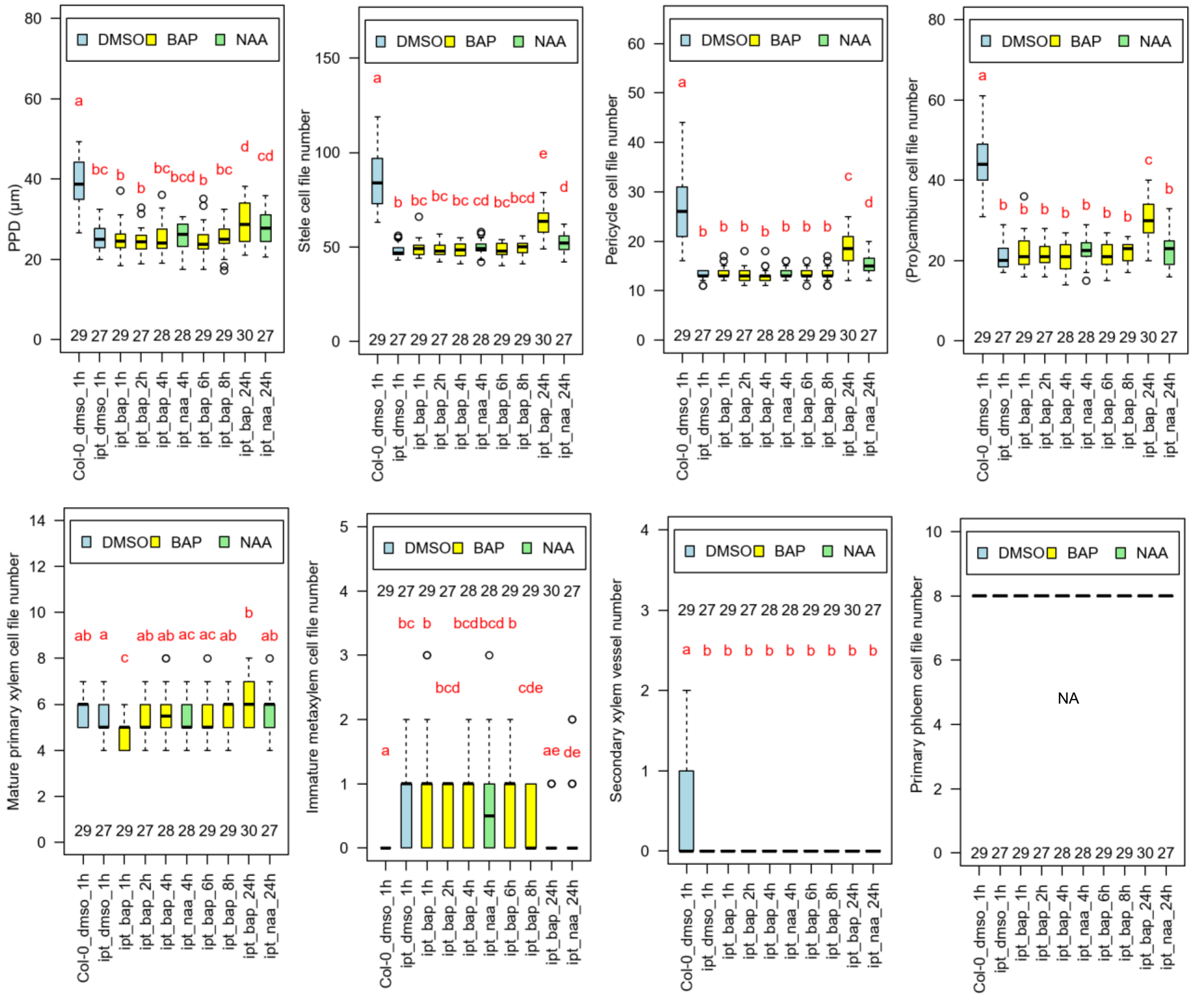

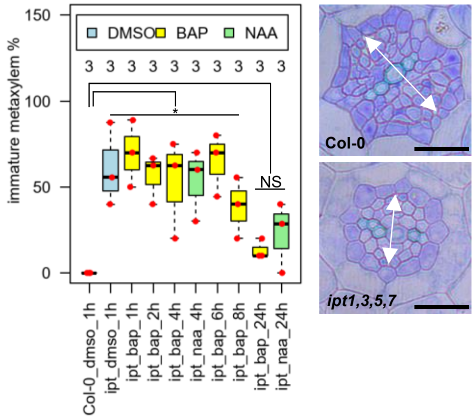

Supplementary Figure S1. Quantification of wild type Col-0 and *ipt1,3,5,7* mutant histology and cell types in response to BAP and NAA time series. Supports Figure 1. Protophloem distance (PPD), stele cell file number, pericycle cell file number, (pro)cambium cell file number, mature primary xylem cell file number, immature metaxylem cell file number, secondary xylem vessel number and primary phloem cell file number were quantified from histological cross sections. Primary phloem cell file number was set to "8" in all samples. Percentage of roots with immature metaxylem was calculated per repeat shown with red dots (n=3). Letters indicate statistical significance following tests with Wilcoxon rank sum method with BH correction (adjusted p-value<0.05), asterisks from pairwise one-tailed Welch t-tests (p<0.05;*). NA=not analyzed, NS=not significant. The numbers depict number of roots analyzed with Fiji (n). The boxes in the box-and-whisker plots show the median and interquartile range, the whiskers indicate 1.5x the interquartile range, and outliers are shown as circles. Representative cross sections from DMSO controls (reused from Fig. 1a) with PPDs (white arrows) are shown. Scale bars=20 µm.

a

Source nodes		Number of edges			
Node	Node_AGI	T1	T2	T3	Total
ABF1	AT1G49720	0	4	0	4
ABF1_1	AT1G49720	0	41	0	41
ANT	AT4G37750	22	3	73	98
ARR7	AT1G19050	0	1	0	1
ASL9	AT1G16530	39	47	0	86
ASL9_1	AT1G16530	47	96	0	143
ASL9_1_2	AT1G16530	53	100	0	153
AT1G19190	AT1G19190	0	1	0	1
AT1G47655	AT1G47655	0	0	156	156
AT1G66020	AT1G66020	1	0	0	1
AT1G68360	AT1G68360	51	3	23	77
AT1G70880	AT1G70880	0	1	0	1
AT1G72920	AT1G72920	0	0	1	1
AT1G72920_1	AT1G72920	0	0	1	1
AT1G77520	AT1G77520	1	1	0	2
AT2G15960	AT2G15960	1	1	0	2
AT2G21188	AT2G21188	0	1	0	1
AT2G26690	AT2G26690	0	0	1	1
AT2G40200	AT2G40200	28	25	0	53
AT2G46690	AT2G46690	1	0	0	1
AT3G16280	AT3G16280	0	0	29	29
AT3G21600	AT3G21600	0	1	0	1
AT3G45680	AT3G45680	0	1	0	1
AT4G13680	AT4G13680	0	1	0	1
AT5G10970	AT5G10970	25	0	0	25
AT5G44390	AT5G44390	0	1	0	1
AT5G52330	AT5G52330	0	1	0	1
AT5G57150	AT5G57150	12	0	0	12
BBX8	AT5G48250	0	23	0	23
CIA2	AT5G57180	0	23	13	36
COL9	AT3G07650	0	24	6	30
CYP71A19	AT4G13290	0	1	0	1
CYP78A9	AT3G61880	0	1	0	1
EBP	AT3G16770	0	25	84	109
ELF4	AT2G40080	1	0	0	1
ESE3	AT5G25190	0	0	63	63
EXL4	AT5G09440	1	0	0	1
GATA5	AT5G66320	0	21	22	43
GDU3	AT5G57685	1	0	0	1
GDU5	AT5G24920	0	1	0	1
HB-12	AT3G61890	8	0	0	8
HB-12_1	AT3G61890	12	0	0	12
IDD2	AT3G50700	17	46	26	89
KIN1	AT5G15960	0	1	0	1
KIN2	AT5G15970	0	1	0	1
LBD4	AT1G31320	0	3	0	3
MYB14	AT2G31180	3	22	0	25
MYB34	AT5G60890	7	0	0	7
MYB77	AT3G50060	0	0	2	2
NAC099	AT5G56620	0	21	81	102
NAC101	AT5G62380	55	0	0	55
PAP2	AT4G29080	0	0	79	79
PCL1	AT3G46640	0	0	3	3
PCL1_1	AT3G46640	0	35	14	49
PCL1_1_2	AT3G46640	0	73	16	89
PMAT2	AT3G29670	1	1	0	2
PRR3	AT5G60100	0	97	21	118
PRR5	AT5G24470	0	22	0	22
SHY2	AT1G04240	0	1	0	1
SVP	AT2G22540	18	10	36	64
SVP_1	AT2G22540	0	10	129	139
TINY2	AT5G11590	10	13	0	23
UMAMIT13	AT2G37450	0	0	1	1
WLIM1	AT1G10200	0	0	17	17
WLIM1_1	AT1G10200	0	3	179	182

b

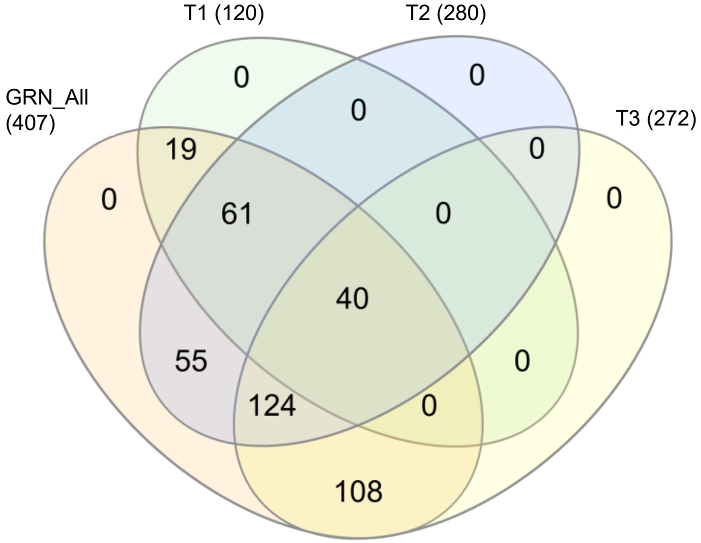

c

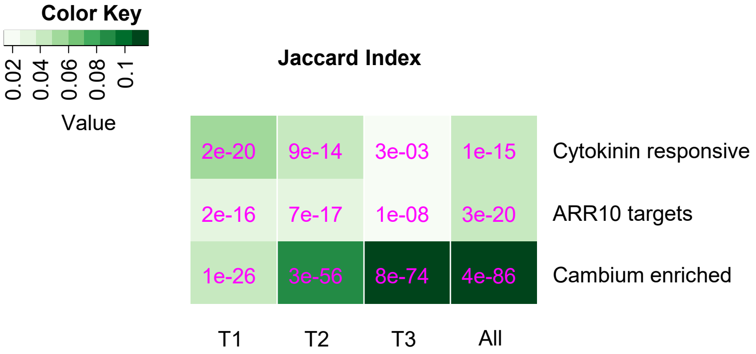

Supplementary Figure S2. Transcriptional cambium activation network. Supports Figure 1. a) The 65 nodes representing probes for 56 transcription factors and the number of their edges in the network at T1, T2, T3 and in total. **b)** Venn diagram of genes clustering with ANT at T1, T2, T3 and in the cambial GRN in total. **c)** GRN genes (T1, T2, T3 and in total ”All” were compared to publicly available gene lists “Cytokinin responsive”, “ARR10 targets” and “Cambium enriched” (adapted from (Bhargava et al. 2013; Zubo et al. 2017; Zhang et al. 2019), respectively). Gene list overlap analysis depicting Jaccard index (green) and Fisher´s exact test with BH-adjusted *p*-value (magenta).

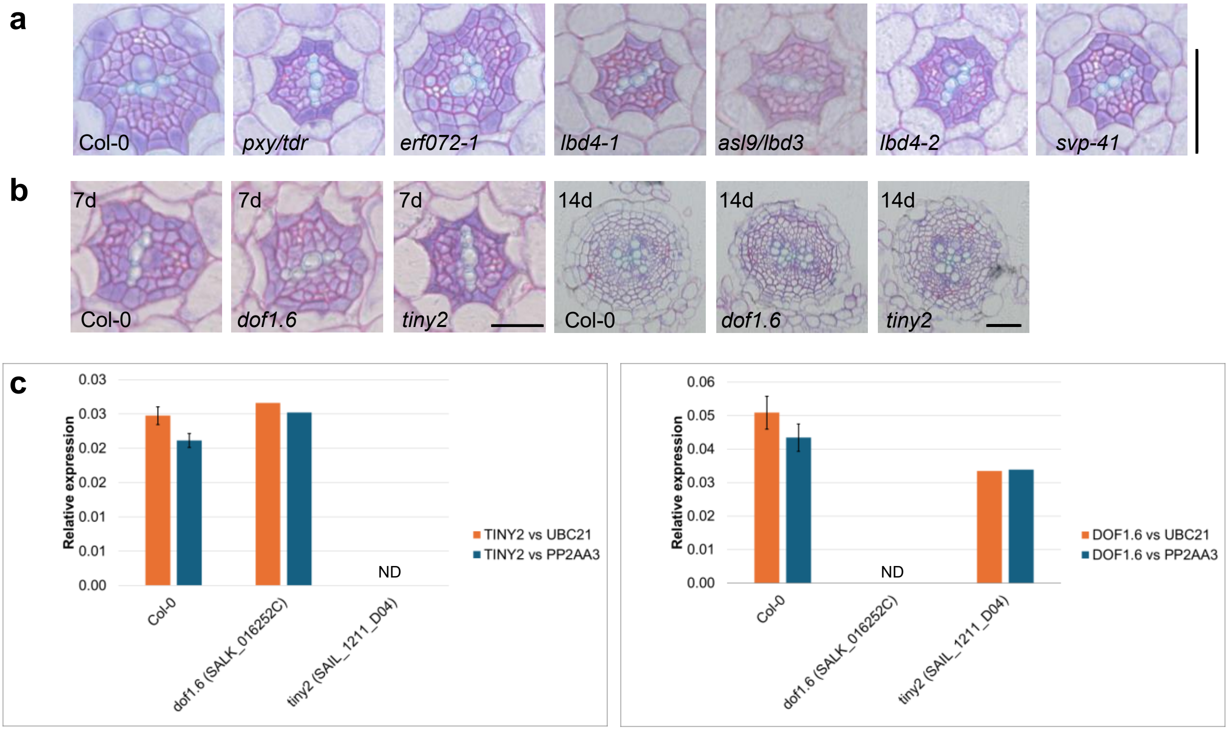

Supplementary Figure S3. Additional information on mutants analyzed in Fig. 1e and Fig 1g. Supports Figure 1. a) Representative cross sections of 7-day-old mutants and Col-0 analyzed in Fig. 1e. Scale bar=50µm. **b)** Representative cross sections of 7-day-old and 14-day-old mutants and Col-0 analyzed in Fig. 1g. Scale bar=20µm. **c)** qPCR analysis of T-DNA insertion lines. 7-day-old Col-0, *tiny2* (SAIL_1211_D04) and *dof1.6* (SALK_016252C) were harvested to analyze the expression of *TINY2* and *DOF1.6* transcripts with qPCR, respectively. Relative expression means ± SD are shown, n=1-2. ND=not detected.

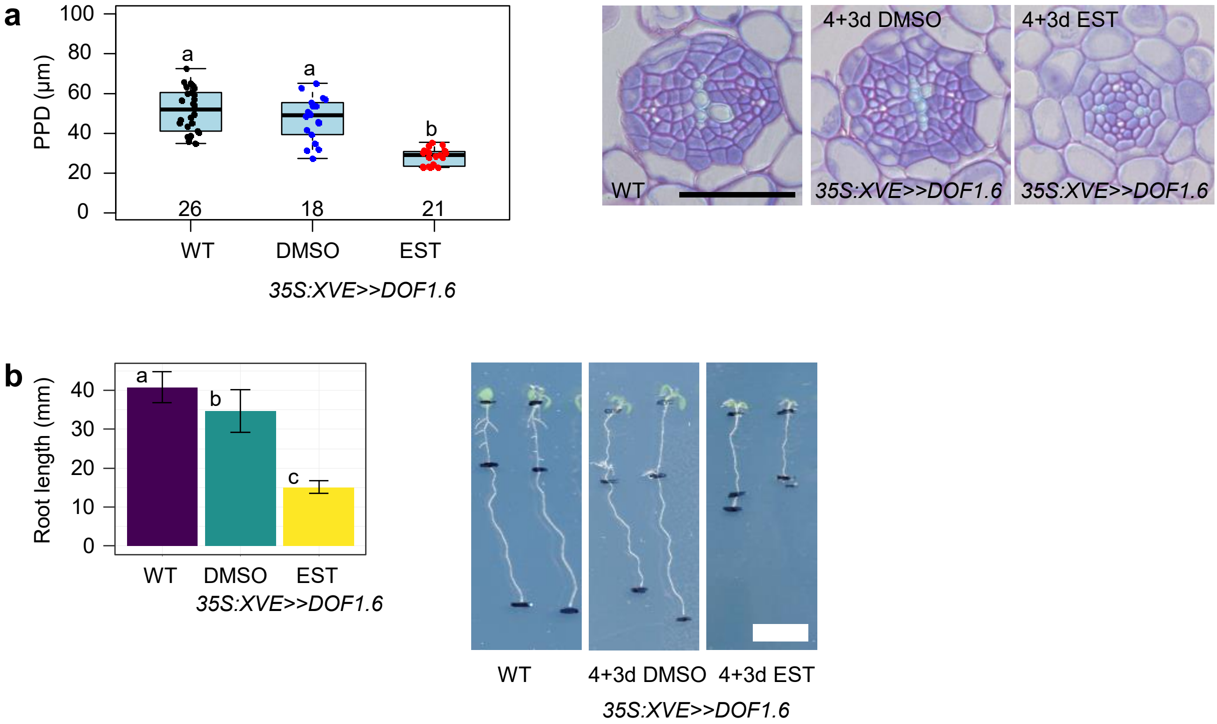

Supplementary Figure S4. DOF1.6 overexpression results. Supports Figure 1. a) Protophloem distance (PPD) of wild type Col-0 (WT) and estradiol-inducible overexpression line *35S:XVE>>DOF1.6* after 4+3 days of DMSO or 5 µM EST. The experiment was repeated thrice. Numbers indicate the amount of histological cross sections (n). Scale bar 50 µm. The boxes in the box-and-whisker plots show the median and interquartile range, the whiskers indicate 1.5x the interquartile range, and outliers are shown as circles. Letters indicate statistical significance following tests with Wilcoxon rank sum method with BH correction (adjusted p-value<0.05). Individual data points are plotted on top of the box-and-whisker plots. **b)** Root primary length was measured from two repeats. Means calculated from 30-40 roots per sample are shown, error bars=standard deviation. Scale bar 1 cm. In a, b, letters indicate statistical groups based on Wilcoxon rank sum test with BH correction (p.adjusted<0.05).

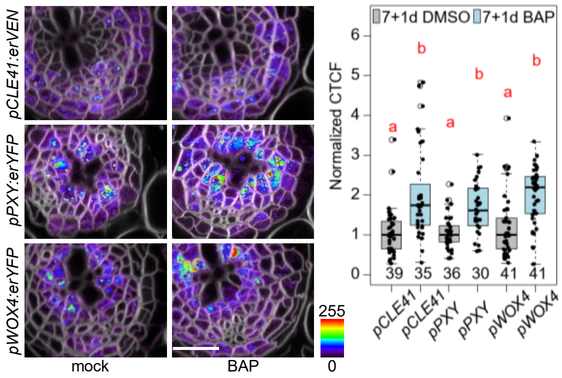

Supplementary Figure S5. Cross sections of CLE41, PXY and WOX4 marker lines after 7+1d DMSO and BAP (1 µM) treatments. Supports Figure 2. Scale bar depicts 10 µm. Color key indicates fluorescence signal scale. Corrected total cell fluorescence was measured in Fiji, and normalized to the median values of DMSO treatments. The experiment was repeated three times. Numbers depict number of roots analyzed with Fiji (n). The boxes in the box-and-whisker plots show the median and interquartile range, the whiskers indicate 1.5x the interquartile range, and outliers are shown as circles. Individual data points are plotted on top of the box-and-whisker plots. Letters indicate statistical significance following Wilcoxon rank sum test with BH correction (adjusted p-value<0.05).

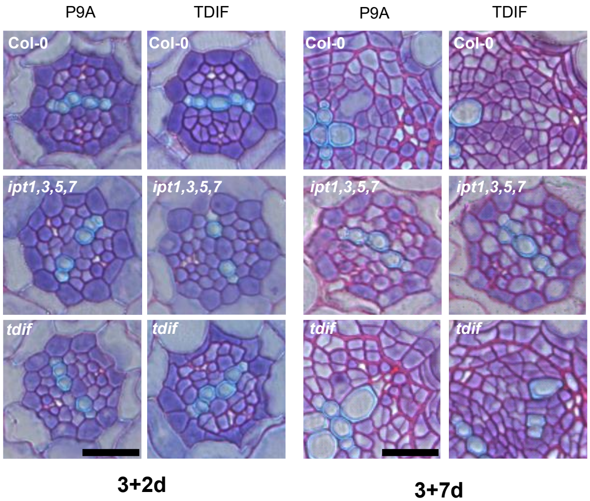

Supplementary Figure S6. Representative cross section images of 3+2d and 3+7d TDIF peptide treatments. Supports Figure 2. Scale bar=20 μm.

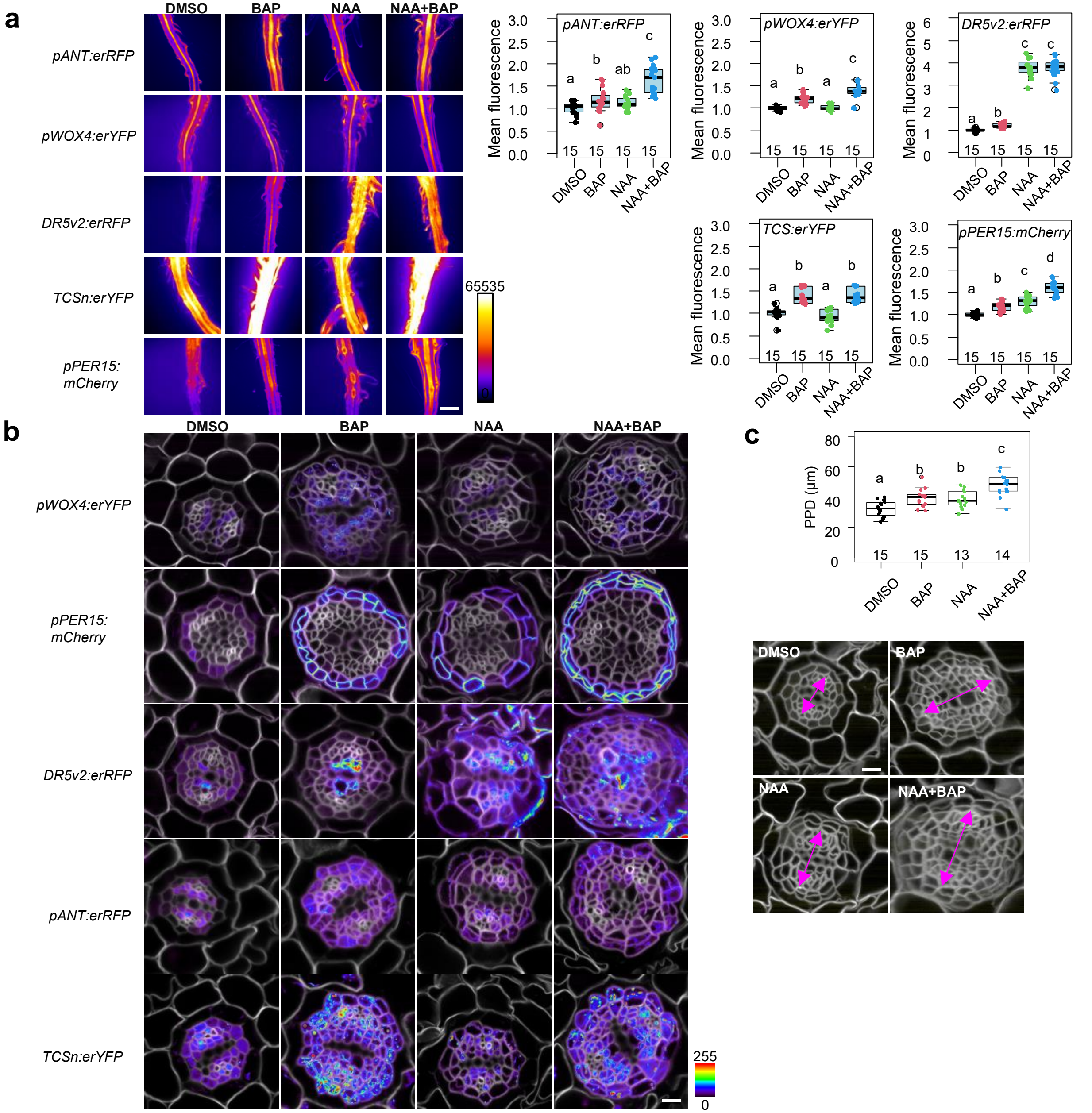

Supplementary Figure S7. Hormone treatments (3+2d) of fluorescent marker lines. Supports Figure 3. a) 3+2 days treatment with DMSO, 1 µM BAP, 1 µM NAA or 1 µM NAA + 1 µM BAP with *pANT:erRFP*, *pWOX4:erYFP*, *DR5v2:erRFP*, *TCSn:erYFP* and *pPER15:mCherry*. Scale bar=500 µm. Fluorescence quantification along the stele was done in Fiji, and average fluorescence from 2 mm length of the root upper part was calculated in R and normalized to the DMSO control average. **b-c)** Representative cross section images of marker line roots (b) and Col-0 (c). Scale bar=10 µm. PPD measurements (arrows) of Col-0 vasculature after 3+2d treatments of DMSO, NAA, BAP or NAA+BAP were done with Fiji. in a, c, numbers depict number of roots analyzed with Fiji (n) in three experiments. The boxes in the box-and-whisker plots show the median and interquartile range, the whiskers indicate 1.5x the interquartile range, and outliers are shown as circles. Individual data points are plotted on top of the box-and-whisker plots. Letters indicate statistical significance following Wilcoxon rank sum test with BH correction (adjusted p-value<0.05). In a, b, color key indicates fluorescence signal scale.

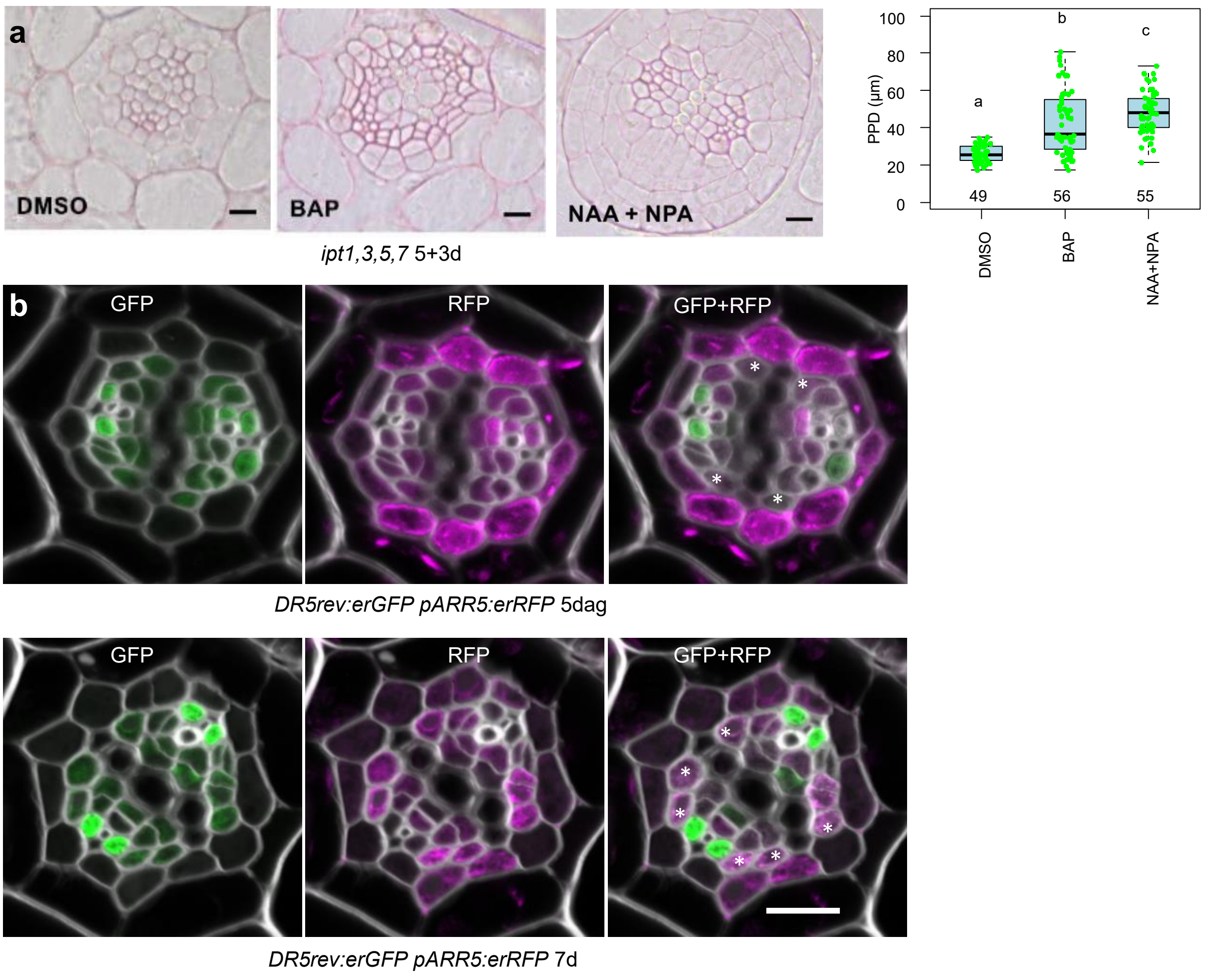

Supplementary Figure S8. Long-term (5+3d) hormone treatments of *ipt1,3,5,7* and additional hormone signaling markers in Col-0 background. Supports Figure 3. a) Three-day auxin (5µM NAA and 20 µM NPA) or cytokinin treatments (5µM BAP) were applied to 5-day-old *ipt1,3,5,7* mutant roots and protophloem distances quantified with Fiji. Scale bar = 10 µm. The experiment was repeated three times. Numbers depict number of roots analyzed (n). The boxes in the box-and-whisker plots show the median and interquartile range, the whiskers indicate 1.5x the interquartile range, and outliers are shown as circles. Individual data points are plotted on top of the box-and-whisker plots. Letters indicate statistical significance following Wilcoxon rank sum test with BH correction (adjusted p-value<0.05). **b)** Cross sections of *DR5rev:erGFP pARR5:erRFP* 5 dag (upper row) and 7 d (lower row). GFP and RFP fluorescence overlap prior to procambial division is marked with asterisk (*). Scale bar represents 10 µm.

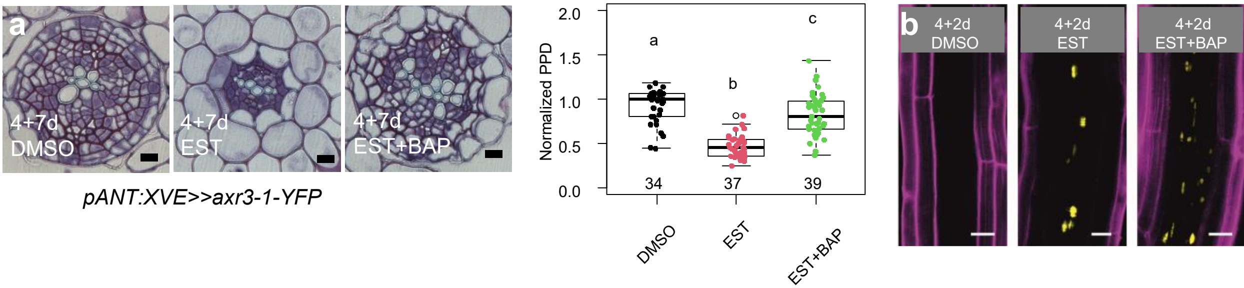

Supplementary Figure S9. Long-term treatment of *pANT:XVE>>axr3-1-YFP* with EST and EST+BAP. **Supports Figure 4. a)** *pANT:XVE>>axr3-1-YFP* grown for 4 days on ½ GM transferred for 7 days onto DMSO, 5 µM EST or 5 µM EST plus 1 µM BAP medium. Scale bar=10 µm. The experiment was repeated three times. Numbers depict number of roots analyzed with Fiji (n). Box-and-whisker plots show PPD normalized to the DMSO control median. The boxes in the box-and-whisker plots show the median and interquartile range, the whiskers indicate 1.5x the interquartile range, and outliers are shown as circles. Individual data points are plotted on top of the box-and-whisker plots. Letters indicate statistical significance following Wilcoxon rank sum test with BH correction (adjusted p-value<0.05). **b)** Longitudinal images of *pANT:XVE>>axr3-1-YFP* grown for 4 days on ½ GM and transferred for 2 days onto DMSO control medium, onto 5 µM β-estradiol medium or onto 5 µM β-estradiol medium plus 1 µM BAP. Scale bar = 25 µm.

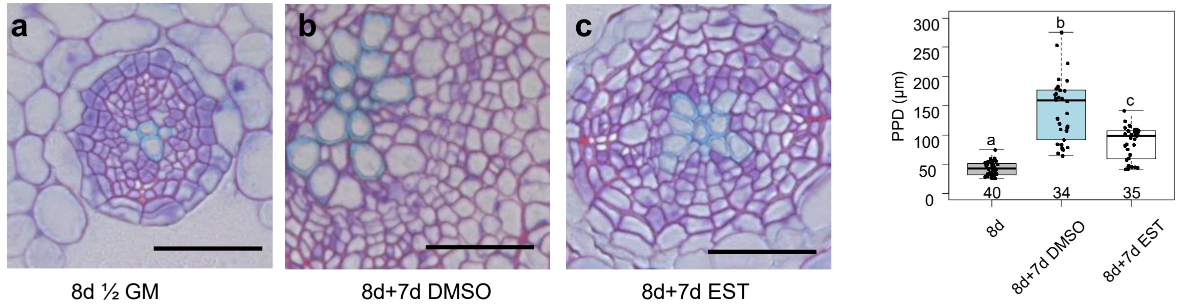

Supplementary Figure S10. Auxin signaling is continuously required for cambium activity. Supports Figure 5. a-c) Histological cross section of *pANT:XVE>>axr3-1-YFP* **(a)** grown for 8 days on ½ GM**, (b)** grown for 8 days on ½ GM and transferred for 7 days onto DMSO control medium or **(c)** 5 µM β-estradiol medium. Scale bar = 50 µm. The experiment was repeated three times. Box-and-whisker plots show PPD and numbers depict number of roots analyzed with Fiji (n). The boxes in the box-and-whisker plots show the median and interquartile range, the whiskers indicate 1.5x the interquartile range, and outliers are shown as circles. Individual data points are plotted on top of the box-and-whisker plots. Letters indicate statistical significance following Wilcoxon rank sum test with BH correction (adjusted p-value<0.05).

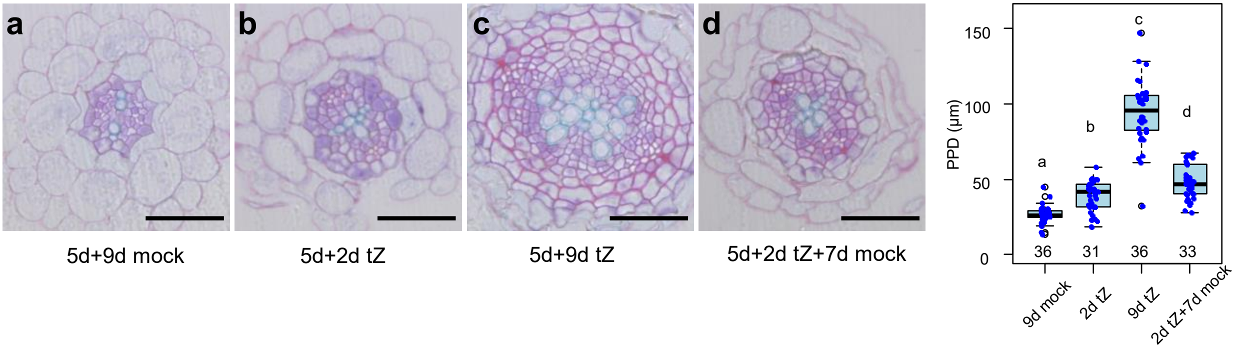

Supplementary Figure S11. Cytokinins are continuously required for root secondary growth. Supports Figure 5. a-d) Histological cross section of *ipt1,3,5,7* grown for 5 days on ½ GM and transferred **(a)** onto mock control medium for 9 days, **(b)** onto 200 nM tZ medium for 2 days, **(c)** onto 200 nM tZ medium for 9 days, and **(d)** onto 200 nM tZ medium for 2 days and transferred back onto mock control medium for 7 days. Scale bar = 50 µm. The experiment was repeated three times. Box-and-whisker plots show PPD and numbers depict number of roots analyzed with Fiji (n). The boxes in the box-and-whisker plots show the median and interquartile range, the whiskers indicate 1.5x the interquartile range, and outliers are shown as circles. Individual data points are plotted on top of the box-and-whisker plots. Letters indicate statistical significance following Wilcoxon rank sum test with BH correction (adjusted p-value<0.05).
